# Effects of Combined CCR5/Integrase Inhibitors-Based Regimen on Mucosal Immunity in HIV-Infected Patients Naïve to Antiretroviral Therapy: A Pilot Randomized Trial

**DOI:** 10.1371/journal.ppat.1005381

**Published:** 2016-01-21

**Authors:** Sergio Serrano-Villar, Talia Sainz, Zhong-Min Ma, Netanya S. Utay, Tae Wook-Chun, Surinder Mann, Angela D. Kashuba, Basile Siewe, Anthony Albanese, Paolo Troia-Cancio, Elizabeth Sinclair, Anoma Somasunderam, Tammy Yotter, Steven G. Deeks, Alan Landay, Richard B. Pollard, Christopher J. Miller, Santiago Moreno, David M. Asmuth

**Affiliations:** 1 University Hospital Ramón y Cajal, Madrid, Spain; 2 University Hospital La Paz, Madrid, Spain; 3 California National Primate Research Center, Davis, California, United States of America; 4 University of Texas Medical Branch, Galveston, Texas, United States of America; 5 Laboratory of Immunoregulation, National Institute of Allergy and Infectious Diseases, Bethesda, Maryland, United States of America; 6 University of California Davis Medical Center, Sacramento, California, United States of America; 7 University of North Carolina School of Medicine, Chapel Hill, North Carolina, United States of America; 8 Rush University, Chicago, Illinois, United States of America; 9 Veterans Administration Northern California Health Care System, Mather, California, United States of America; 10 University of California San Francisco, San Francisco, California, United States of America; Emory University, UNITED STATES

## Abstract

Whether initiation of antiretroviral therapy (ART) regimens aimed at achieving greater concentrations within gut associated lymphoid tissue (GALT) impacts the level of mucosal immune reconstitution, inflammatory markers and the viral reservoir remains unknown. We included 12 HIV- controls and 32 ART-naïve HIV patients who were randomized to efavirenz, maraviroc or maraviroc+raltegravir, each with fixed-dose tenofovir disoproxil fumarate/emtricitabine. Rectal and duodenal biopsies were obtained at baseline and at 9 months of ART. We performed a comprehensive assay of T-cell subsets by flow cytometry, T-cell density in intestinal biopsies, plasma and tissue concentrations of antiretroviral drugs by high-performance liquid chromatography/mass spectroscopy, and plasma interleukin-6 (IL-6), lipoteichoic acid (LTA), soluble CD14 (sCD14) and zonulin-1 each measured by ELISA. Total cell-associated HIV DNA was measured in PBMC and rectal and duodenal mononuclear cells. Twenty-six HIV-infected patients completed the follow-up. In the duodenum, the quadruple regimen resulted in greater CD8^+^ T-cell density decline, greater normalization of mucosal CCR5^+^CD4^+^ T-cells and increase of the naïve/memory CD8^+^ T-cell ratio, and a greater decline of sCD14 levels and duodenal HIV DNA levels (P = 0.004 and P = 0.067, respectively), with no changes in HIV RNA in plasma or tissue. Maraviroc showed the highest drug distribution to the gut tissue, and duodenal concentrations correlated well with other T-cell markers in duodenum, i.e., the CD4/CD8 ratio, %CD4^+^ and %CD8^+^ HLA-DR^+^CD38^+^ T-cells. Maraviroc use elicited greater activation of the mucosal naïve CD8^+^ T-cell subset, ameliorated the distribution of the CD8^+^ T-cell maturational subsets and induced higher improvement of zonulin-1 levels. These data suggest that combined CCR5 and integrase inhibitor based combination therapy in ART treatment naïve patients might more effectively reconstitute duodenal immunity, decrease inflammatory markers and impact on HIV persistence by cell-dependent mechanisms, and show unique effects of MVC in duodenal immunity driven by higher drug tissue penetration and possibly by class-dependent effects.

## Author Summary

Despite the efficacy of ART to suppress HIV replication in blood, HIV-infected individuals experience persistent immunologic dysfunction and inflammation that predict mortality and have been related to a chronically injured gut-associated lymphoid tissue. These gut abnormalities results in a ‘leaky gut’, from which microbial antigens are translocated into the bloodstream and contribute to a pathogenic vicious circle of inflammation and viral persistence. The effects of first-line ART in gut tissue remain largely unexplored.

Herein we show that restoration of mucosal immune abnormalities following ART initiation might depend upon gut tissue penetration and could be affected by initiating ART with a combined CCR5 and integrase inhibitors-based regimen. Our findings describe potential beneficial consequences of combined CCR5 and integrase inhibitor-based first-line ART regimens and highlight the impact of MVC on mucosal immunity, particularly in the duodenum, that may be due to a higher tissue penetration and by virus-independent mechanisms mediated by class-dependent effects. These findings suggest an opportunity for targeting mucosal immune dysfunction and chronic inflammation with regimens designed to impact within lymphatic tissues, and provide rationale for expanding the armamentarium available to target the gut in future HIV reservoir reduction strategies.

## Introduction

A widely accepted conceptual model of HIV immunopathogenesis recognizes how the gut might serve as a sanctuary for HIV replication and a portal of systemic inflammation, likely contributing to residual morbidity observed in most cohorts of treated HIV-infected individuals [1–4]. SIV/HIV targets the gut at many different levels, including immunological, structural and microbial. Immunologically, there is a rapid and massive depletion of CD4^+^ T-cells, in part related to the presence in mucosal tissues of levels of activated CD4^+^ T-cells expressing the main HIV receptor CCR5 [5]. Known structural effects include the loss of epithelial barrier function [5–9]. Microbial changes characterized as bacterial dysbiosis have been described with shifts in microbial distributions and metabolic activity towards pathways associated with activation of the innate and adaptive immunity [10–13]. Ultimately, these disturbances are believed to result in a ‘leaky gut’, from which microbial antigens are translocated into the bloodstream and contribute to a sustained pro-inflammatory state in treated HIV patients. Biomarkers of inflammation correlate strongly with the risk of mortality even after fully achieving HIV suppression and CD4^+^ T-cell restoration [1,2,14].

There is now increasing awareness that ART might not be able to fully normalize health, and clinical research is shifting beyond strategies aimed at suppressing HIV replication and normalizing peripheral CD4^+^ T-cell counts, to evaluation of the impact of novel strategies designed to reduce bacterial translocation and inflammation. However, these abnormalities have proven to be difficult to reverse, likely due to the multifaceted pathways involved in sustained immune activation, increased inflammation and the residual morbidity seen in treated patients such as metabolic and cardiovascular disease [2,15]. So far, ART initiation during acute HIV infection is most effective in blunting systemic and mucosal immune dysfunction, although does not fully normalize GALT defects [16,17]. However, the timing of ART initiation necessary to achieve such outcomes is a narrow window within weeks of HIV transmission. Such early initiation of ART is not always possible, so other strategies for gut immune, structural/functional and microbial reconstitution are an important unmet medical need. Although data on concentrations of antiretroviral drugs in lymphatic tissues (the gut and lymph nodes) are limited, it has been shown that suboptimal concentrations are achieved for some agents within these sites to fully suppress HIV replication, where virus production persists despite otherwise effective ART [18,19]. These findings provide a rationale for testing the effects of ART combinations with better penetration in lymphatic tissues on gut immunity.

We hypothesized that the restoration of mucosal immune abnormalities following ART initiation might depend upon gut tissue penetration and could be affected by initiating ART with a combined CCR5 and integrase inhibitor-based regimen. We performed a study where we randomized HIV-infected individuals to three different first-line ART regimens: efavirenz (EFV), maraviroc (MVC) or maraviroc and raltegravir (MVC+RAL), each in combination with tenofovir disoproxil fumarate/emtricitabine (TDF/FTC), and we included a group of healthy controls. These regimens were selected specifically due to the observed impact of C-C chemokine receptor type 5 (CCR5) inhibition by MVC on CD4 count rise, decline in immune activation and favorable penetration into GALT [20–24] and preliminary data suggesting a favorable effect of RAL on microbial translocation, and penetration and HIV persistence in GALT [25–28]. Upper endoscopy and flexible sigmoidoscopy were performed at baseline and after nine months of treatment to obtain duodenal and rectal biopsies. A comprehensive assessment of a large panel of parameters reflective of T-cell reconstitution in peripheral blood, rectum and duodenum, markers of HIV persistence, plasma markers of immune activation and microbial translocation, and concentrations of antiretroviral drugs in each compartment were measured to explore potential correlates of ART regimen effectiveness in immune reconstitution.

## Methods

### Study design

This pilot, randomized clinical trial enrolled HIV-infected patients naive to ART and a cohort of healthy volunteers who were similar to the HIV-infected patients in age and lifestyle at UC Davis Medical Center between November 2009 and August 2012. Patients had to have CD4^+^ T-cell counts above 50 cells/mm^3^ within 30 days of screening and CCR5 tropism by Trofile ES. Exclusion criteria were safety parameters related to undergoing upper endoscopy with biopsies (grade II anemia or abnormal coagulation parameters were excluded). Patients were randomized to either EFV, MVC or MVC plus RAL (1:1:1), each in combination with a fixed-dose combination of TDF/FTC. Within-class changes were allowed in the event of side effects to the non-nucleoside reverse transcriptase inhibitor (NNRTI). Patients who required ART changes to another class outside of their randomized assignment or who were lost to follow-up are not included in this analysis. In controls, blood and tissue samples were obtained only at baseline and no longitudinally measurements were performed.

The Investigational Pharmacy Service at UC Davis Medical Center generated a random sequence for the clinical design. These were provided to the study team in closed envelopes per their protocol for randomized clinical trials. After a subject was found to be eligible for enrollment based on screening laboratory criteria and viral tropism assay results, they were scheduled for the initiation visit, which consisted of the upper and lower endoscopy. At the completion of that visit, an independent study coordinator was asked to select the next envelope and inform the team of the cohort allocation assignment. A single study nurse coordinator was responsible for recruitment, study procedure scheduling, sample collection, and treatment dispensing. The study nurse coordinator was the contact for all study activities but was not involved in the randomization sequence number generation or assignment to study cohort as stated above. The study drugs were then prescribed based on that assignment. No blinding to treatment allocation was implemented.

### Ethics statement

The research protocol was approved by the UC Davis Institutional Review Board and all participants provided written informed consent prior to initiation of study procedures. Clinical Trial Registry Number Identifier (Clinicaltrials.gov): NCT00870363.

### Measurements

Blood was collected by sterile venipuncture using ethylenediamine tetraacetic acid (EDTA) as an anticoagulant. Rectal biopsies were obtained via flexible sigmoidoscopy at 10–15cm from the anal verge as previously described [29]. Duodenal biopsies were obtained via upper endoscopy. Rectal and duodenal biopsies were either placed immediately in liquid nitrogen, paraformaldehyde for paraffin-embedding or processed for single cell suspension following collagenase digestion as previously described [30]. Peripheral blood mononuclear cells (PBMC) were isolated from blood using Ficoll-Hypaque (Pfizer- Pharmacia, New York, NY).

The single-cell suspensions were stained with Aqua-viability dye and QuantumDot655 anti-CD45RA (clone MEM-56) from Invitrogen (Carlsbad, California, USA); PacBlue-anti-CD3 (clone UCHT1) and fluorescein isothiocyanate-anti-human leukocyte antigen-DR (clone L243) from Biolegend (San Diego, California, USA); ECD-anti-CD4 (clone SFCI12T4D11) from Beckman- Coulter (Brea, California, USA); and PE-anti-CD38 (clone HB7), PE-Cy7-anti-CCR7 (clone 3D12), and APC-H7-anti-CD8 (clone SK1) from Becton-Dickinson (San Jose, California, USA) according to manufacturers’ recommendations. PBMCs were processed with the intestinal cells in identical fashion. Fluorescence-activated cell sorter (FACS) analysis was performed on a custom Becton-Dickinson LSR II flow cytometer used for data acquisition and analyzed with FlowJo (TreeStar, Ashland, Oregon, USA). Gating strategy included using an FMO (fluorescence-minus-one) to determine the cut-off for positive cells for CCR7, CD38, and HLA-DR for each run. A representative example of the gating strategy for the lymphocyte maturational subsets is shown in (S1 Fig). Combinations of markers were calculated in FlowJo, using the Boolean gate function. Lymphocyte maturational subsets are defined as naive (CD45RA+/CCR7+), central memory (CD45RA-/CCR7+), effector memory (CD45RA-/CCR7-), or RA+ memory (CD45RA+/CCR7-) [31]. T-cells with an activation phenotype are defined as co-expression of HLA-DR and CD38 on respective lymphocyte population. Rainbow beads (Spherotec, Lake Forest, Illinois, USA) from a single lot were used to calibrate each photomultiplier tube to a uniform gain to insure stability in signal between samples. An additional control standard peripheral blood sample aliquoted and cryopreserved at the beginning of the clinical trial was run in parallel at the time each sample was analyzed to further assess run to run variability.

IFA analysis was performed as we have previously reported [25]. Briefly, the primary antibodies used were polyclonal anti-CD3 rabbit serum (Dako Inc., Carpinteria, California, USA) and monoclonal anti-CD4 or CD8 mouse serum (Leica Micreosystems, Buffalo Grove, Illinois, USA). Binding of CD3 and CD4 or CD8 receptors were detected simultaneously using Alexafluor 488-labeled polyclonal goat antirabbit IgG (Molecular Probes, Eugene, Oregon, USA) and Alexafluor 568- labeled polyclonal goat antimouse IgG (Molecular Probes). The numbers of positive cells were counted by a single observer (Z-MM) and presented as cells/mm^2^ of lamina propria or intraepithelial regions (above the basement membrane) of duodenal mucosa [32–34]. Representative sections are presented in S2 Fig.

Plasma was assessed by immunoassay for IL-6 (high-sensitivity kit, R&D Systems), sCD14 (R&D Systems), CRP (CardioPhase high-sensitivity kit,-CRP assay, Siemens), lipoteichoic acid (LTA) and zonulin-1 (ALPCO) levels.

To determine the frequency of CD4^+^ T-cells carrying HIV-1 DNA in infected individuals, In order to determine the frequency of cells carrying HIV DNA, DNA was digested from cell pellets (1 x 10^6 PBMCs or intestinal single cell suspension) with XbaI and 500ng of DNA was subjected to PCR (Bio-Rad Laboratories) according to the manufacturers specifications. The amplification reaction was carried out using HIV-specific primers and probe and RPP30 (housekeeping gene)-specific primers and probe. The following primers were used for amplification of HIV LTR:5GRAACCCACTGCTTAAGCCTCAA-3 (5 primer) and 5TGTTCGGGCGCCACTGCTAGAGA-3 (3 primer) along with the fluorescent probe 5FAM-AGT_AGT_GTG/ZEN/TGC_CCG_TCT_GTT-IB-IABkFQ-3. The following primers will be used for amplification of RPP30: 5-GATTTGGACCTGCGAGCG-3 (5 primer) and 5-GCGGCTGTCTCCACAAGT-3 (3 primer) along with the fluorescent probe 5-HEX-TTCTGACCTGAAGGCTCTGCGC-IABkFQ-3. PCR conditions consisted of a denaturation step at 95°C for 3 min, followed by 45 cycles of 15 sec at 95°C and 1minat 59°C. Serially diluted ACH-2 DNA (40,000, 8000, 1600, 320, 64, 12.8, 2.56, and 0.56 cell equivalents per well in triplicates) was also subjected to the PCR conditions above to obtain standard curves. The detection limit of the assay was 2.56 copies of HIV DNA.

For detection of unspliced form of HIV-1 RNA, total RNA was isolated from plasma and tissue by using RNeasy Mini Kit (Qiagen), according to the manufacturers specifications. Synthesis of cDNA was performed using qScript Flex cDNA kit (Quanta Biosciences) with 1g of RNA and HIV specific primer (US 3) 5-TCT_CCT_TCT_AGC_CTC_CGC_TAG_TC-3 in total volume 20 mcL. cDNA (4 mcL) was then used for amplification according to the manufacturers specifications. The following primers and probe were be used: 5-TCT_CTA_GCA_GTG_GCG_CCC_GAA_CA-3 (5 primer), 5-TCT_CCT_TCT_AGC_CTC_CGC_TAG_TC-3 (3 primer), and 5-6FAM-CAA_GCC_GAG_TCC_TGC_GTC_GAG_AG-IB-IABkFQ-3 (probe) [35]. HIV copy numbers were determined per 2μg of RNA from tissue samples and normalized by duodenal CD4+ T cell numbers determined by IFD.

### Measurement of plasma and tissue drug concentrations

Drug concentrations were quantified by LC-MS/MS analysis using methods similar to those previously published [28,36,37] Tissue biopsies were homogenized in 1mL of 70:30 acetonitrile-1mM ammonium phosphate (pH 7.4) with a Precellys 24 tissue homogenizer (Bertin Technologies, Montigny-le-Bretonneux, France). Plasma underwent protein precipitation with an organic solution containing a stable-labeled intlysis. For all compounds, a Shimadzu high-performance liquid chromatography system was used for separation, and an AB SCIEX API 5000 mass spectrometer (AB SCIEX, Foster City, CA, USA) equipped with a turbo spray interface was used as the detector. Samples were analyzed with a set of calibration standards (0.02 to 20 ng/mL for tissue and 5–5,000ng/mL for plasma) and quality control (QC) samples. The precision and accuracy of the calibration standards and QC samples were within the acceptable range of 15%. To convert volume to mass, tissue density was assumed to be 1.06 g/cm3. All LC-MS/MS data underwent QC by a designated individual not directly involved in this study to ensure accuracy. Two subjects in the NNRTI arm switched from EFV to NVP. Given this low number of samples from patients treated with NPV, we did not compare NVP concentrations with the other drugs. MVC concentrations could not be measured in one subject in the MVC arm and RAL concentrations could not be measured in one subject in the quadruple regimen arm, due to insufficient tissue sample.

### Statistical methods

Qualitative variables were reported as a frequency distribution whereas quantitative variables were described as median and interquartile ranges (IQRs). Cross-sectional pairwise comparisons between groups were performed using the non-parametric Kruskal-Wallis and Mann-Whitney U tests. The Spearman rank correlation coefficient was used to analyze the correlation between continuous variables. We used linear mixed models with a random effect for each patient to allow for correlations caused by repeated observations to assess whether longitudinal changes in numerical outcome measures were overall significantly different from baseline. A robust variance estimator was used given the limited sample size and the deviations from normality. Interaction terms were created to assess whether these changes over time differed significantly between treatment groups. The equality of all mean changes among the three groups was tested using a Wald test for the interaction term and is referred as “Overall differences” across the text and Figs. Continuous outcome variables were log-transformed when necessary to satisfy model assumptions. All study time-points were included in the linear mixed models, although for clarity, only month 0 and 9 month measurements are plotted for blood measurements in the Figs. Results are expressed as estimated means (95% CI) unless stated otherwise. A *P* value of ≤ 0.05 was considered statistically significant in this pilot study seeking new relationships between simultaneous immunologic, virologic, pharmacologic, and biomarker parameters across three compartments. All statistical analyses were conducted with Stata v. 13.0 (StataCorp LP College Station, Texas, USA).

## Results

**Fig 1:**
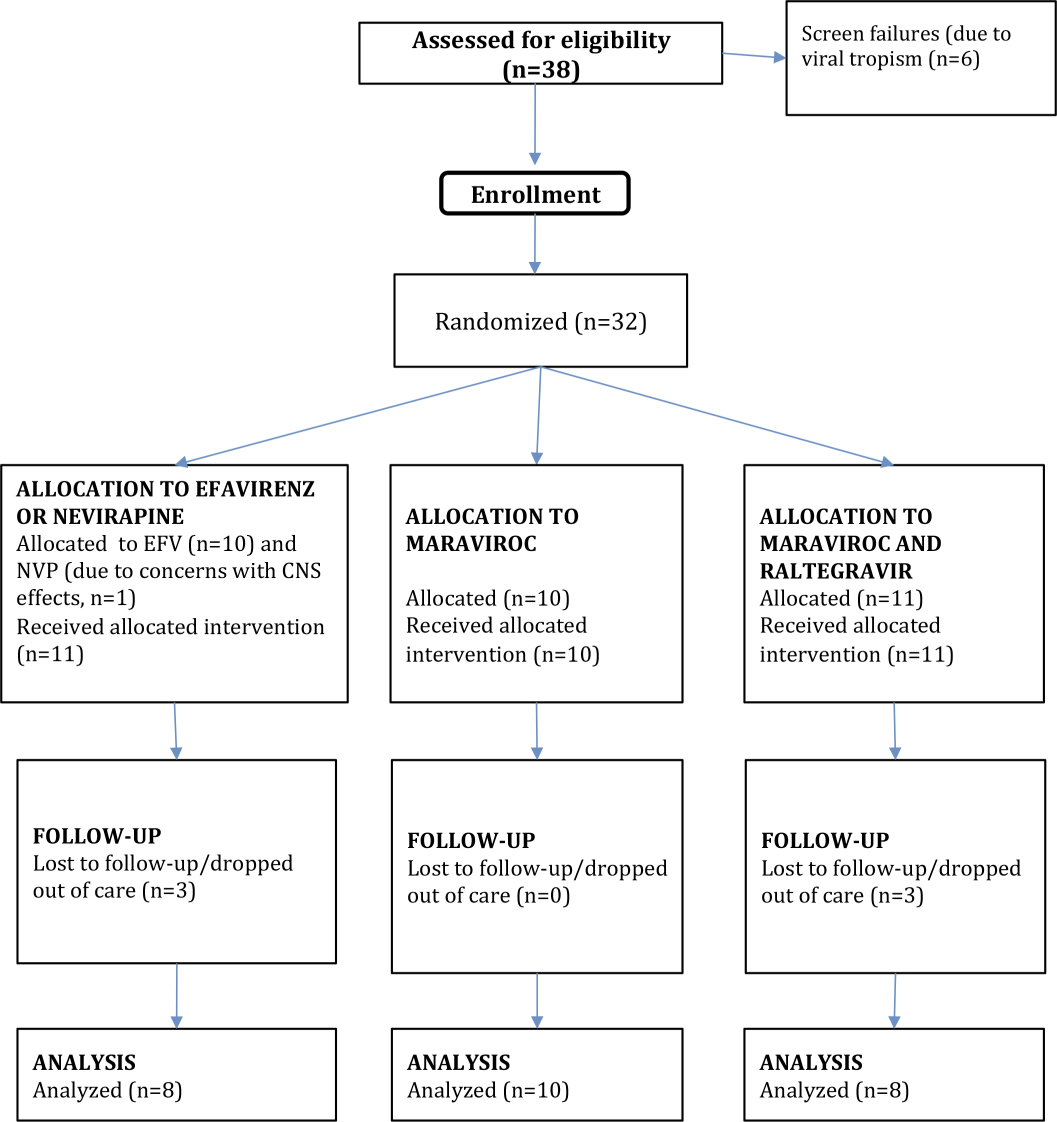
Study profile.

Thirty-two HIV-infected patients underwent randomization and baseline procedures. Twelve HIV-uninfected healthy controls similarly were enrolled. Six patients were excluded from the analyses due to loss of follow-up (n = 5) or change in ART to another class (protease inhibitors, n = 1). Twenty-six patients completed the 9 months of treatment, 8 with NNRTI (7 on EFV and 1 on nevirapine), 10 with MVC and 8 with MVC+RAL (Fig 1). Median age was 37 years (25, 42). Median baseline CD4^+^ T-cell counts were 322 cells/mm^3^ (233, 536), 441 (369, 511) and 453 (334, 713), respectively (between-group comparisons, *P* = not significant (NS)). We observed statistically significant increases of peripheral CD4^+^ T-cell counts in all groups: 151 cells/mm^3^ (43, 386), 239 (128, 288) and 281 (120, 342) with NNRTI, MVC and MVC+RAL, respectively (between-group comparisons, *P* = NS). All individuals achieved complete HIV suppression. General characteristics of study participants are summarized in Table 1.

**Table 1:** General characteristics of study participants.

	HIV-uninfected	HIV-infected		
	n = 12	n = 26		
		EFV	MVC	MVC+RAL
		n = 8	n = 10	n = 8
**Age (years, IQR)**	35 (29, 42)	37 (25, 46)	38 (25, 43)	35 (27, 40)
**Male gender (No., %)**	7 (58%)	8 (100%)	7 (70%)	7 (87.5%)
**Race (No., %)**				
Asian	1 (8.3%)	1 (12.5%)	0 (0%)	1 (12.5%)
Black	1 (8.3%)	1 (12.5%)	5 (50%)	4 (50%)
Caucasian	9 (55.0%)	4 (50%)	3 (30%)	3 (35.5%)
Pacific Islander	1 (8.3%)	1 (12.5%)	0 (0%)	0 (0%)
Hispanic	0 (0%)	1 (12.5%)	2 (20%)	0 (0%)
**CD4** ^**+**^ **T-cell count (cells/mm** ^**3**^ **, IQR)**	NA	322 (233, 536)	441 (369, 511)	453 (334, 713)
**CD4/CD8 ratio (IQR)**	1.6 (1.2, 2.6)	0.33 (0.25, 0.66)	0.41 (0.31, 0.51)	0.59 (0.49, 0.78)
**HIV RNA Level (log**_**10**_ **copies/mL)**	NA	4.2 (3.7, 5.2)	4.3 (3.6, 4.6)	4.3 (4.1, 4.8)

### The intervention elicited distinct effects in lymphocyte subsets across compartments

#### Effects on peripheral and mucosal CD4+ T-cells and CD4/CD8 ratio

**Fig 2:**
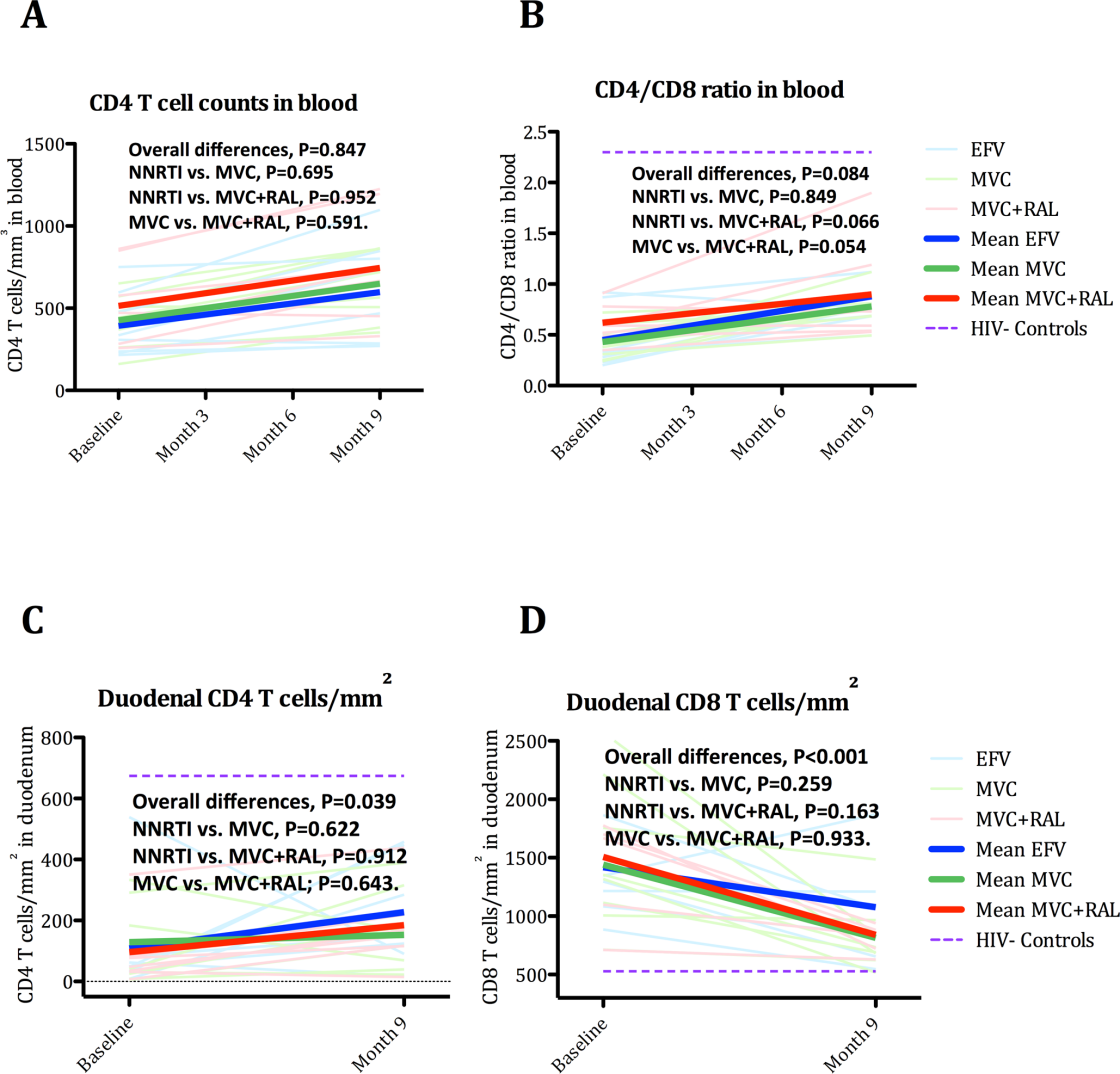
Changes in peripheral and duodenal CD4^+^ and CD8^+^ T-cells. Panel A. Changes in peripheral CD4^+^ T-cell counts. Panel B. Changes in peripheral CD4/CD8 ratio. Panel C. Changes in duodenal CD4^+^ T-cell density. Panel D. Changes in duodenal CD8^+^ T-cell density. The purple dash line represents cross-sectional mean values in controls. The equality of all mean changes among the three groups was tested using a Wald test for the interaction term and is referred as “Overall differences”. A statistically significant interaction term (group*month) identified significantly different slopes between treatment groups.

During treated HIV infection, both changes of CD4^+^ T-cell counts and persistence of a low CD4/CD8 ratio predict mortality [38,39]. We first analyzed variations of CD4^+^ T-cell counts and CD4/CD8 ratio in blood (see Fig 2A and 2B and S1 Table), and no differences were detected between groups. Similarly, no differences in changes of %CD4^+^ T-cells or CD4/CD8 ratio were found in GALT between cohorts (see S2 Table for rectum, and S3 Table for duodenum).

#### Inhibition of CCR5 receptor elicited greater reduction of duodenal CD8^+^ T-cell density

IFA analysis revealed that compared with MVC, both NNRTI and MVC+RAL experienced greater improvement of CD4^+^ T-cell density in duodenum (median delta CD4^+^ T-cells/mm^2^, 24, 119 and 89; respectively; P = 0.039). In contrast, compared with NNRTI, both MVC and MVC+RAL provided similar efficacy to further reduce duodenal CD8^+^ T-cell infiltration (median delta CD8^+^ T-cells/mm^2^, -290, -522 and -679; respectively; P<0.001)(see S3 Table and Fig 2C and 2D). The fact that a greater CD8^+^ T-cell decline was appreciated in both MVC-based regimens argues that inhibition of CCR5 receptor might better decrease duodenal CD8^+^ T-cell infiltration and/or a reduction in the antigen stimulus that drives CD8 homing to this compartment.

#### Inhibition of CCR5 receptor enhanced CD8^+^ T-cell differentiation in the gut

**Fig 3:**
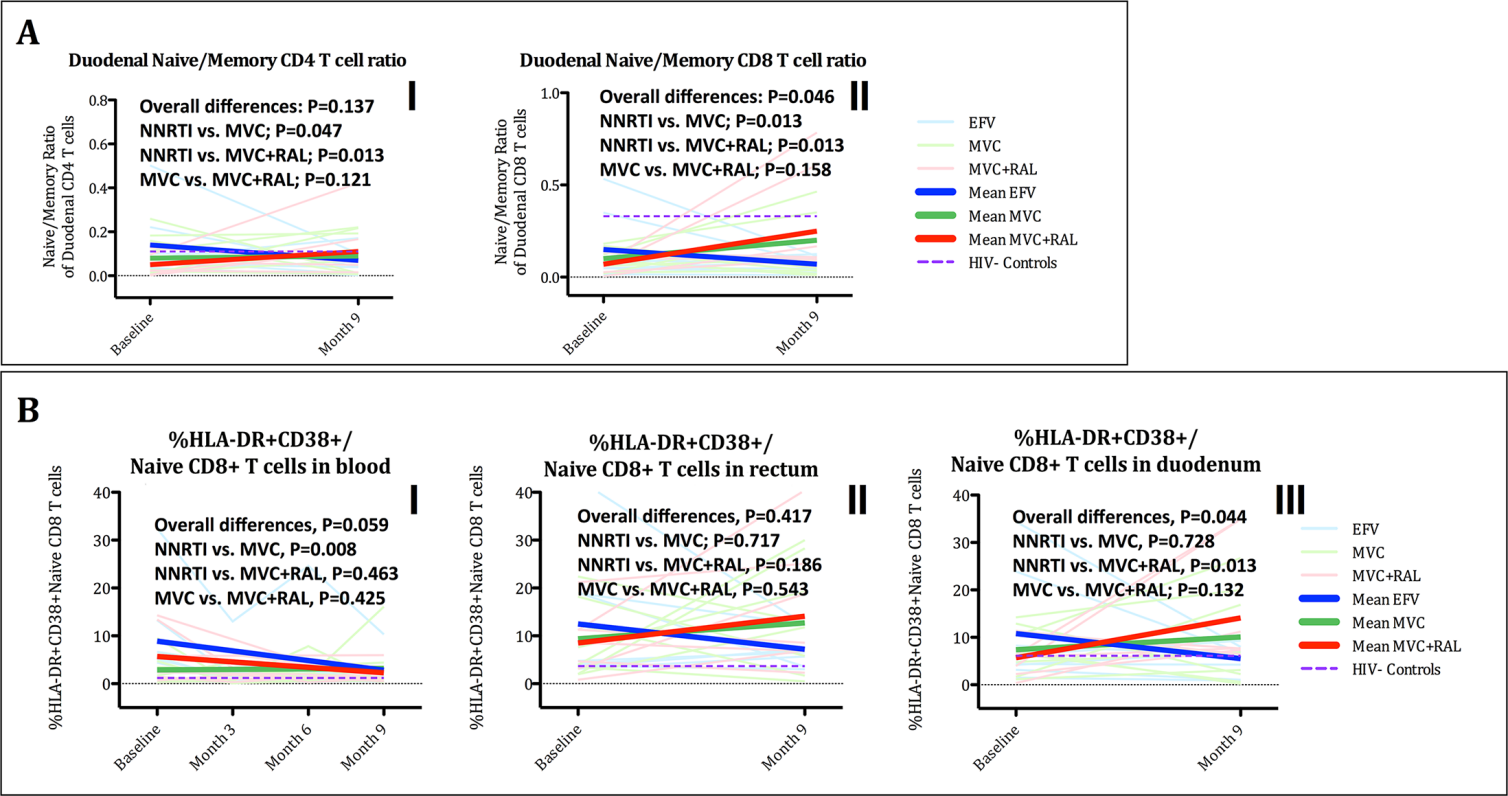
Changes in the naive/memory ratio and CD38^+^HLA-DR^+^ naive CD8 T-cells in duodenum. Panel A. Changes in the naïve/memory ratio in duodenum for CD4^+^ (I).and CD8^+^ T-cells (II). Panel B. Changes in %HLA-DR^+^CD38^+^ */*naive CD8 T-cells in blood (I), rectum (II) and duodenum (III). The purple dash line represents cross-sectional mean values in controls. The equality of all mean changes among the three groups was tested using a Wald test for the interaction term and is referred as “Overall differences”. A statistically significant interaction term (group*month) identified significantly different slopes between treatment groups.

Chronic HIV infection is characterized by expansion of activated (HLA-DR^+^CD38^+^) and skewed differentiation of T-cells from naïve towards effector memory. These phenomena appear more apparent within the CD8^+^ T-cell subset, translate a loss of naïve cells and an aberrant accumulation of long-lived and dysfunctional memory T cells, and are increasingly recognized as a hallmark of HIV-mediated chronic immune dysfunction [40,41]. Hence, we examined changes in activated T-cells and maturational (naïve/memory) subsets (see S1–S3 Tables) [42]. As expected, T-cell activation significantly decreased in blood, rectum and duodenum, but no significant differences between treatment groups were detected. Of maturational subsets, the naïve/memory ratio was lower at baseline compared to controls and increased for both CD4^+^ and CD8^+^ T-cells in blood (see S1 Table) and rectum (see S2 Table) in all three cohorts. We observed different effects however, in the duodenum. Compared to controls, CD8^+^ T-cell differentiation was dramatically impaired in HIV-infected participants, as reflected by a markedly lower CD8^+^ naïve/memory ratio (see S3 Table and Fig 3A). While with NNRTI, the naïve/memory ratio still decreased during the study for both CD4^+^ and CD8^+^ T-cells, these ratios increased with MVC, and still more with MVC+RAL (NNRTI vs. MVC, P = 0.008 and 0.013, respectively; NNRTI vs. MVC+RAL, P = 0.013 and 0.013, respectively, and MVC vs. MVC+RAL, P = 0.158 and 0.132, respectively). These findings indicate that while all three first-line strategies might achieve comparable improvements of T-cell activation in blood and rectum, MVC, within the triple or quadruple regimen, could elicit greater amelioration of T-cell differentiation in duodenum compared with NNRTI-based regimens.

#### Inhibition of CCR5 receptor resulted on paradoxical activation of mucosal naive CD8^+^ T-cells that correlated with improvement of T-cell differentiation

**Fig 4:**
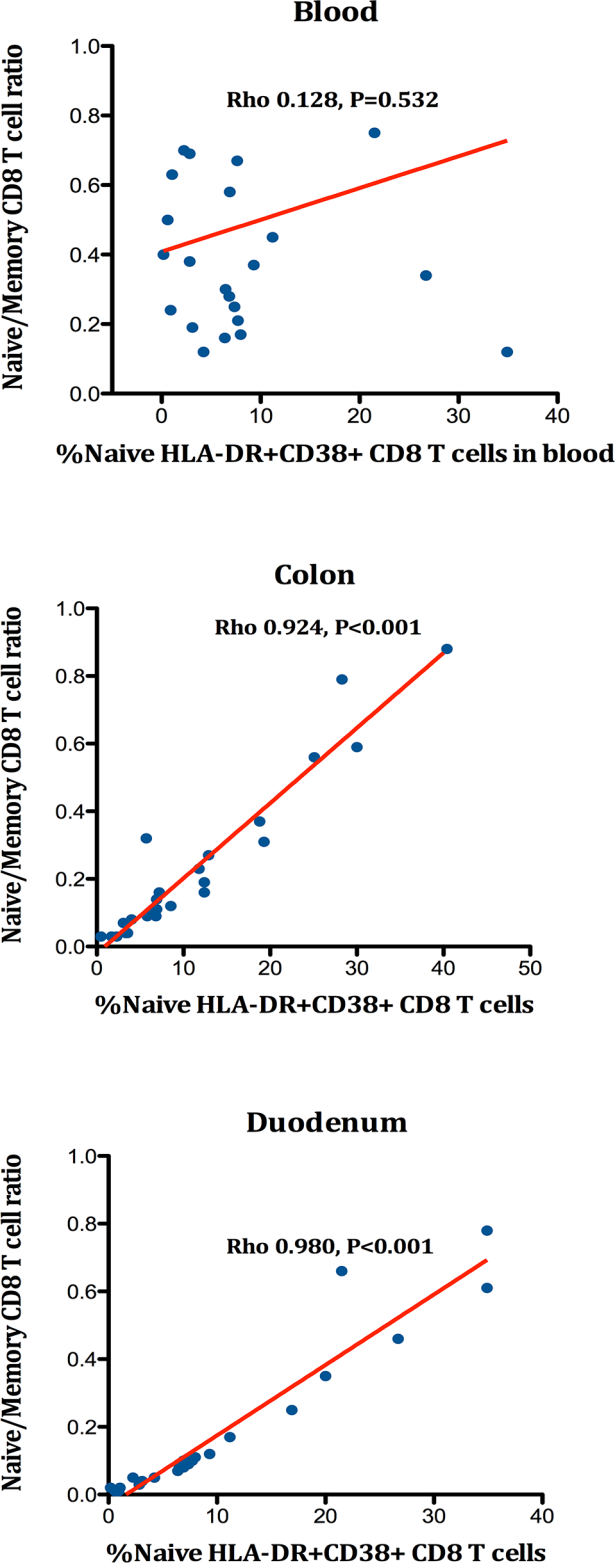
Correlations between the %naive-activated CD8^+^ T-cells and the naïve/memory CD8 T-cell ratio in blood, rectum and duodenum.

The subset of naïve activated T-cells is considered of special importance because this population represents the first step of immune response upon antigenic stimulation that ultimately yields to T-cell expansion and generation of the effector response [43–45]. Intriguingly, while in the grouped analysis the %HLA-DR^+^CD38^+^ naïve CD4^+^ and CD8^+^ T-cells tended to reach normal levels in blood following suppressive treatment (see S1 Table and Fig 3B), we observed opposite effects between NNRTI and MVC in rectum and duodenum. Whereas the naive CD8^+^ T-cells with an activated phenotype decreased towards normal concentrations with NNRTI, these numbers increased in rectum and duodenum within the MVC and MVC+RAL cohorts (Overall differences in rectum, P = 0.417; in duodenum, P = 0.044). This difference reached statistical significance for the pairwise comparison of NNRTI vs. MVC+RAL in the duodenum (P = 0.013), but not between MVC vs. MVC+RAL (P = 0.132) (see S2 and S3 Tables and Fig 3B). This pattern of distinct changes between groups was not appreciated in the memory/activated CD8^+^ T-cell pool (all comparisons, P = ns.). To further understand the potential immunological implications of this phenomenon, and given the parallelism with the effects observed on the naïve/memory CD8^+^ T-cell ratio, we calculated the correlations between the naive/activated CD8^+^ T-cells and the naïve/memory CD8^+^ T-cell ratio. We observed a strong positive correlation in both tissue compartments (All Rho>0.90, P<0.001) that were not observed in the peripheral compartment (see Fig 4). These findings suggest a possible paradoxical effect of MVC on T-cell activation of the naïve subset in GALT that seems directly attributable to MVC rather than to the use of a quadruple regimen. This unexpected increase of T-cell activation positively influenced T-cell differentiation by preserving the naïve T-cell subset in the gut mucosa and may actually reflect a restoration of T-cell homeostasis that is critical to normal mucosal immune function [5].

#### Combination CCR5 and integrase inhibitors-based ART elicited greater normalization of percentage of CD4^+^ mucosal T-cells expressing CCR5

**Fig 5:**
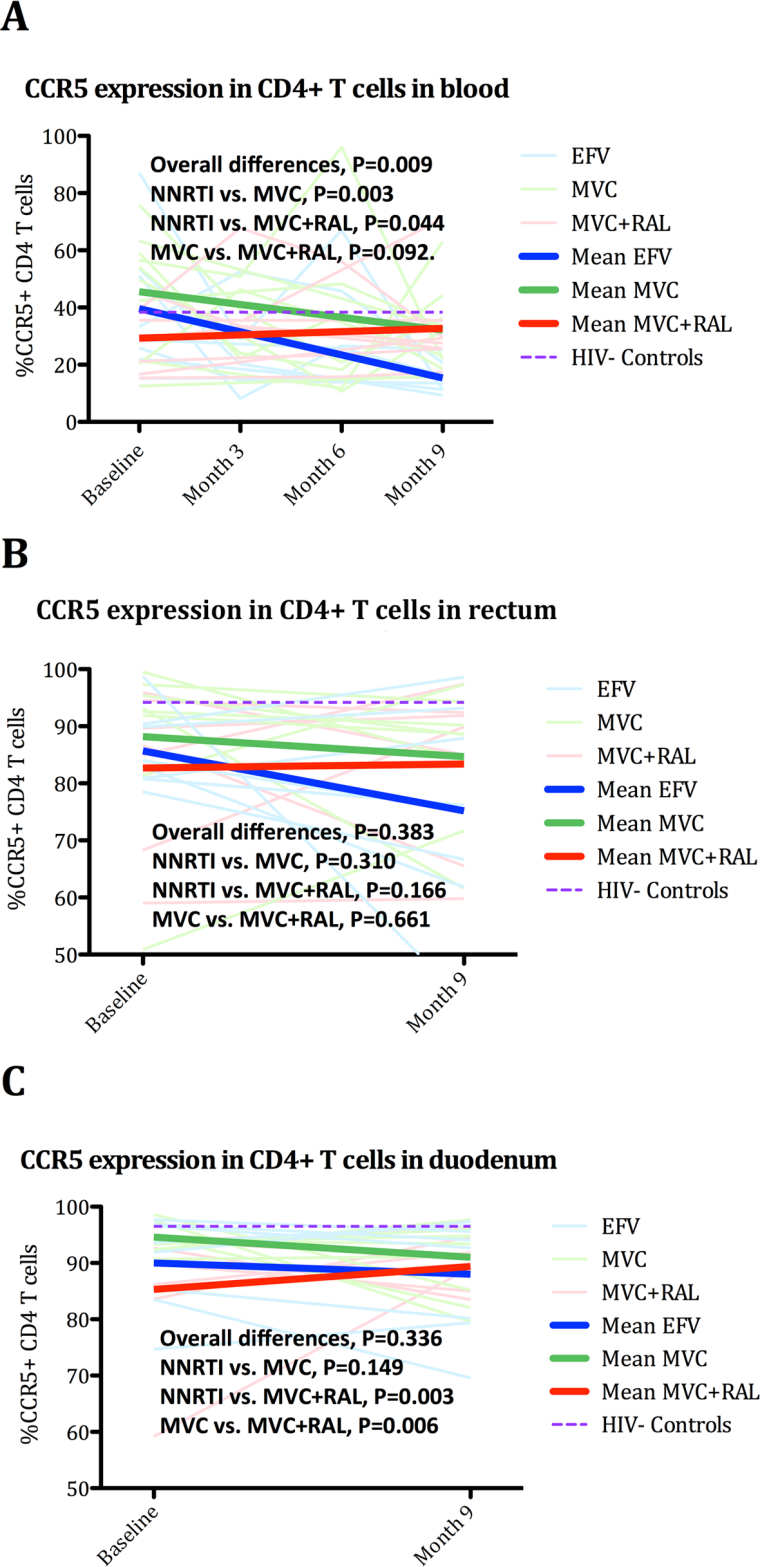
Changes in %CCR5 T-cells in blood, rectum and duodenum. Panel A. Changes in %CD4^+^CCR5^+^ T-cells in blood. Panel B. Changes in %CD4^+^CCR5^+^ T-cells in rectum. Panel C. Changes in %CD4^+^CCR5^+^ T-cells in duodenum. The Purple dash line represents cross-sectional mean values in controls. The equality of all mean changes among the three groups was tested using a Wald test for the interaction term and is referred as “Overall differences”. A statistically significant interaction term (group*month) identified significantly different slopes between treatment groups.

Intestinal mucosa contains a high concentration of HIV-susceptible CCR5^+^ CD4^+^ T-cells that are preferentially depleted early in acute infection [46]. Early depletion of CCR5-expressing CD4^+^ T-cells appears to contribute to persistent inflammation and a permissive state for microbial antigen translocation from the gut into the bloodstream [1–3]. Compared to controls, CD4^+^/CCR5^+^ T-cells were depleted at baseline in all groups, in rectum and duodenum, but not in blood (HIV^-^ vs. HIV^+^ participants, in blood, 35.8 [26.2, 42.2] vs. 35.5% [21.4, 53.6], P = 0.891; in rectum 95% [92.7, 98] vs. 89.8 [81.2, 93.8], P = 0.017; and in duodenum 99% [96.7, 99.3] vs. 93.4 [88% vs. 94.7%], P = 0.0009) (see Fig 5A and 5C). During follow-up, we observed a unique pattern of CD4^+^/CCR5^+^ T-cell changes across all compartments in the MVC+RAL cohort. While subjects in either the NNRTI or MVC cohorts experienced further CD4^+^/CCR5^+^ T-cell loss in blood, rectum and duodenum, the MVC+RAL cohort showed improvement of these cells in all three compartments, with the differences being more apparent in duodenum (NNRTI vs. MVC+RAL, P = 0.003 and MVC vs. MVC+RAL, P = 0.006). Hence, ART initiation with a MVC+RAL containing regimen resulted on better CD4^+^/CCR5^+^ T-cell restoration of the gut, particularly in the duodenum. Since no such improvement was observed in the MVC group, these data suggested that the effect might be attributable to the combination of CCR5 and integrase-inhibitor regimen.

### The quadruple ART regimen elicited a greater reduction of cell associated HIV DNA levels in duodenum

**Fig 6:**
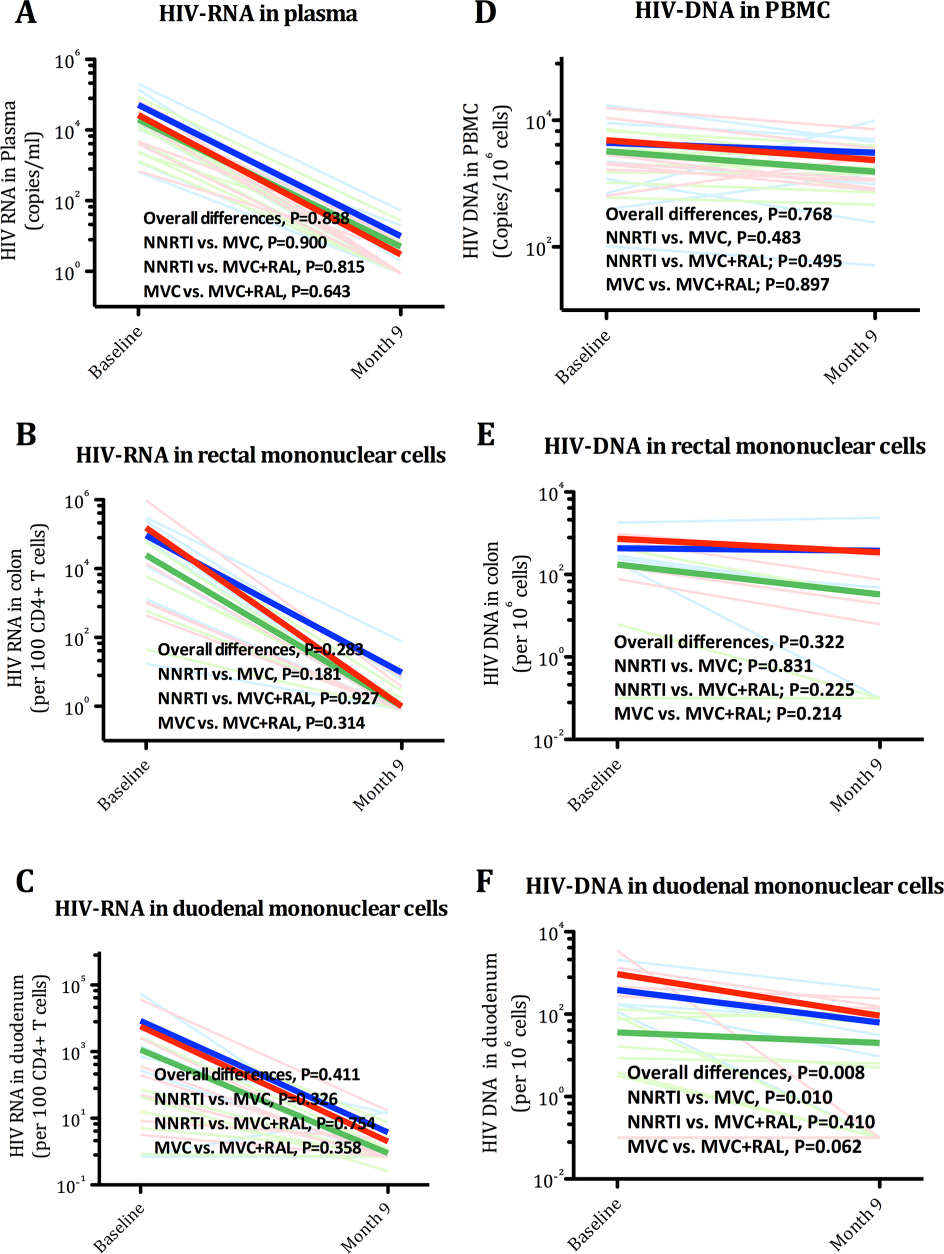
Effects of three ART regimens on markers of viral persistence. Panel A. HIV RNA in plasma. Panel B. Cell-associated HIV RNA in rectum. Panel C. Cell-associated HIV RNA in duodenum. Panel D. Cell-associated HIV DNA in PBMC. Panel E. Cell-associated HIV DNA in rectum. Panel F. Cell-associated HIV DNA in duodenum. The equality of all mean changes among the three groups was tested using a Wald test for the interaction term and is referred as “Overall differences”. A statistically significant interaction term (group*month) identified significantly different slopes between treatment groups.

There is increasing recognition that the gut might serve as a sanctuary for HIV persistence. Reduced penetration or increased metabolism of antiretroviral drugs in tissue compartments and persistence of bacterial antigen stimulation could locally induce pro-inflammatory cytokines known to enhance cell cycling and/or HIV replication. These conditions could ultimately promote residual viral replication and persistence [47]. We hypothesized that a regimen including four drugs might lead to greater suppression of new rounds of viral replication and reduce residual HIV to a higher degree than a regimen with fewer drugs. We then analyzed changes in plasma low-level HIV RNA and cell-associated HIV RNA in tissue, and cell-associated HIV DNA in blood and tissue (see S4 Table and Fig 6). In duodenum, we detected significant differences among treatment groups in duodenum (P = 0.008) in HIV DNA, that appeared driven by a greater decay of HIV DNA levels with MVC+RAL compared with MVC (P = 0.062). These differences, however, were not associated with changes in plasma HIV RNA or in cell-associated HIV RNA in gut tissue, indicating that the greater reduction of HIV DNA levels observed in duodenum in the quadruple regimen was driven by mechanisms independent of viral suppression.

### The distinct effects on duodenal T-cells observed within MVC arms are related to higher drug distribution to the gut

**Fig 7:**
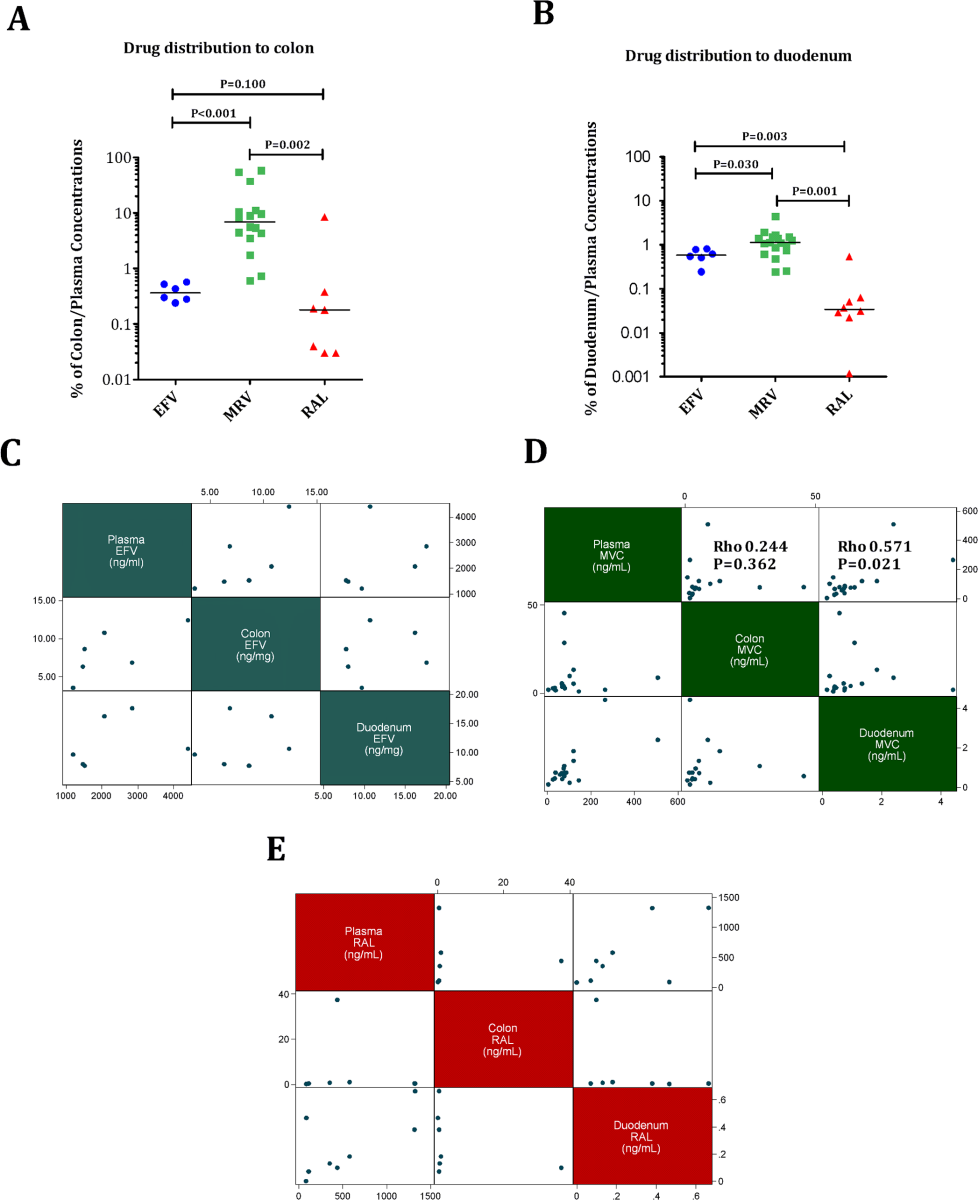
Tissue drug distribution. Panels A-B. Percentages of rectum/plasma (A) and duodenum/plasma (B) drug concentrations. Maraviroc reached the highest distribution to rectum and duodenum (all P<0.005). Panel C-E. Correlations between plasma, rectum and duodenal levels of EFV (C), MVC (D) and RAL (E). MVC plasma levels correlated better with tissue levels than RAL or EFV. The Purple dash line represents cross-sectional mean values in controls.

To explore whether the observed treatment effects could be dependent upon tissue penetration or, instead, be driven by pharmacological class-specific effects, we determined the concentrations of efavirenz/nevirapine, MVC and RAL at each site, and calculated the percentage of the concentrations per mg of tissue over the concentrations per ml of blood. MVC showed the highest distribution to rectum and duodenum, and the differences reached statistical significance (see Fig 7A and 7B). We used linear regression models to estimate tissue concentrations for each drug based on plasma concentrations, and only plasma MVC concentrations were predictive of duodenal concentrations (β = 0.001, P = 0.004) (see Fig 7C and 7E).

**Fig 8:**
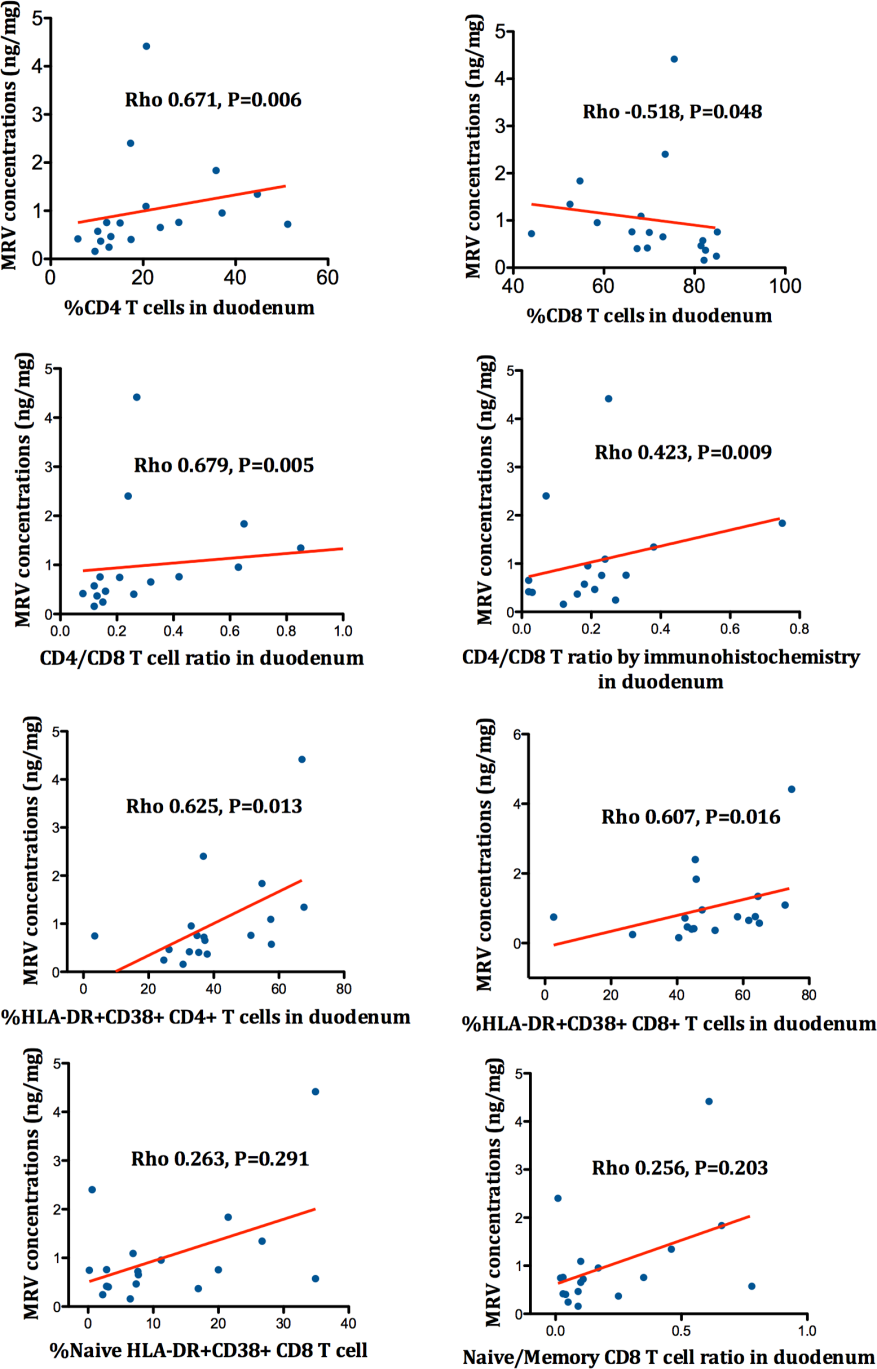
Correlations between MVC duodenal concentrations and several markers of duodenal immunity.

To further understand whether this higher penetration of MVC into the gut mucosa might lead to detectable local immunological effects, we calculated the correlations between drug concentrations and T-cell markers for each compartment. In duodenum, concentrations of MVC correlated well with a number of duodenal T-cell populations, i.e., CD4^+^ and CD8^+^ T-cell percentage (Rho 0.671, P = 0.006 and Rho -0.518, P = 0.048, respectively), CD4/CD8 ratio (Rho 0.679, P = 0.005), and CD4^+^ and CD8^+^ T-cells with the activated phenotype (Rho 0.625, P = 0.013 and Rho 0.607, P = 0.016, respectively)(see Fig 8). No similar correlations were observed for RAL or NNRTI concentrations in rectum or duodenum, which might be related to the limited distribution of these drugs to tissues, or to a lower statistical power to detect associations given the lower number of subjects receiving RAL or NNRTI than MVC.

**Fig 9:**
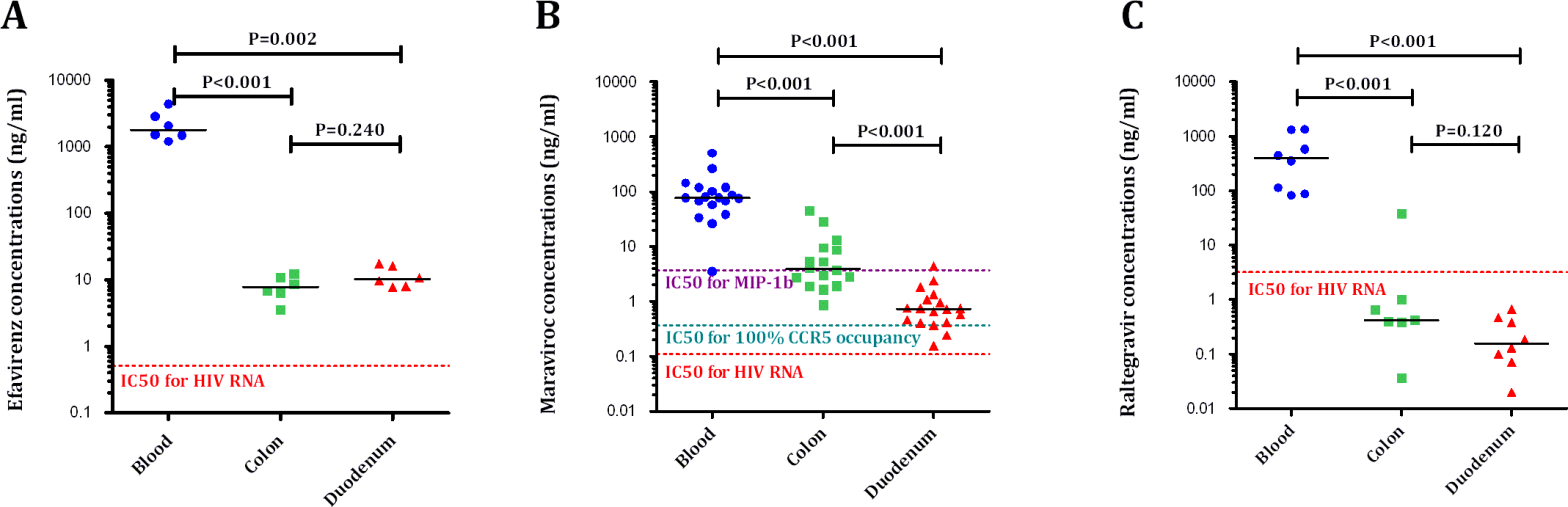
Absolute concentrations in each compartment of efavirenz (A), maraviroc (B) and raltegravir (C) related to the IC50 for HIV-1 replication.

Then, we compared the concentrations of each drug by gut site in order to understand whether drug penetration accounts for the observed immune reconstitution between the randomized cohorts, we compared the concentrations of each drug by gut site related to their IC50 for HIV-1 replication [48–50](see Fig 9). Only MVC concentrations were observed to have any differences between the compartments, with higher median values in rectum than in duodenum [2.1 ng/ml (3.9–9.5) vs. 0.4 (0.7–1.2), P<0.001), respectively], being the concentrations in this latter compartment below the IC50 for the CCR5 ligand MIP-1β inhibition. These findings suggest that the unique effects observed in duodenum with MVC were driven by bystander mechanisms rather than by a better drug penetration.

### Inhibition of CCR5 receptor impacted on greater decline of sCD14 levels and quadruple ART induced greater increase of zonulin-1 levels

**Fig 10:**
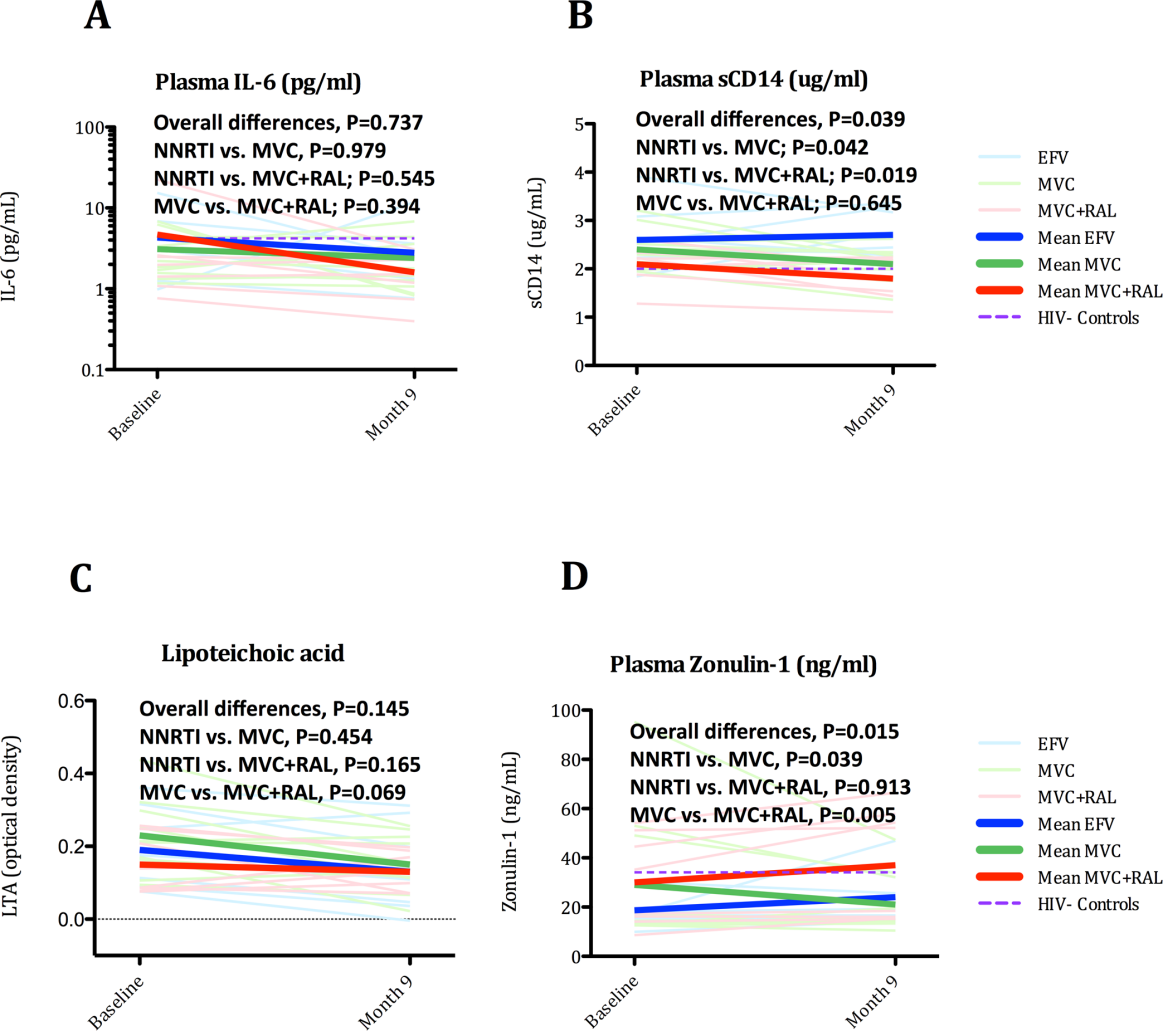
Effects of three ART regimens on markers of innate immunity. Panel A. Changes in plasma IL-6. Panel B. Changes in plasma sCD14. Panel C. Changes in plasma LTA. Panel D. Changes in plasma zonulin-1. The Purple dash line represents cross-sectional mean values in controls. The equality of all mean changes among the three groups was tested using a Wald test for the interaction term and is referred as “Overall differences”. A statistically significant interaction term (group*month) identified significantly different slopes between treatment groups.

Sustained systemic immune activation during treated HIV infection is associated with increased pro-inflammatory biomarkers and is now recognized as an important factor contributing to excess mortality in virologically suppressed treated patients. Hence, we assessed the effects of each regimen on plasma markers of inflammation (interleukin- 6 (IL-6)), monocyte activation (soluble CD14 (sCD14)), bacterial translocation (lipoteichoic acid (LTA)), and epithelial barrier function (zonulin-1). Changes during treatment and the effects of each treatment arm are summarized in S5 Table and Fig 10. In an analysis combining all groups, levels of IL-6, sCD14 and LTA significantly decreased from baseline to month 9 of treatment [IL-6, from 2.2 pg/ml (1.4, 4.3) to 1.6 (0.9, 2.8), P = 0.059; sCD14, from 2.3 ug/ml (2.0, 2.5) to 2.2 (2.0, 2.3), P = 0.038 and LTA from 0.17 (0.09, 0.25) to 0.13 (0.09, 0.20) P = 0.006], while zonulin-1 levels did not change [from 17.3 ng/mL (14.2, 35.6) to 17.5 (14.9, 37.3)]. In the grouped comparison, MVC+RAL showed the greatest decline of sCD14 (see Fig 10B) (P = 0.039) and the greatest increase of zonulin-1 (see Fig 10D) (P = 0.015). For sCD14 levels, the MVC vs. NNRTI comparison was significant (P = 0.019), but not the MVC vs. MVC+RAL comparison (P = 0.645), indicating that this effect was likely attributable to MVC, rather than a consequence of the quadruple regimen. In contrast, for zonulin-1, the MVC vs. MVC+RAL comparison was significant (P = 0.005), suggesting that the effect was attributable to the quadruple regimen or, alternatively, to raltegravir. To further understand these findings, we analyzed the relationships of sCD14 and zonulin-1 levels with drug concentrations (see S6 Table). Levels of sCD14 correlated well with duodenal concentrations of MVC (Rho -0.671, P = 0.004), but zonulin-1 levels did not (Rho 0.209, P = 0.438), supporting the concept that the larger decrease of sCD14 observed in both MVC-based regimens is probably a consequence of a higher concentration of this drug in gut tissue or its effect on immune restoration in GALT leading to reduced bacterial antigen stimulation systemically. In addition, plasma raltegravir, but not tissue concentrations, correlated well with zonulin-1 levels, arguing that the greater increase observed with MVC+RAL compared with MVC might be driven by the use of a fourth drug rather than by raltegravir tissue penetration.

## Discussion

In this randomized clinical trial, we found evidence that the four-drug regimen including both CCR5 and integrase inhibitors might more effectively reconstitute duodenal immunity. The data also suggested, although less clearly, that a quadruple regimen including MVC+RAL could impact on HIV tissue levels. Furthermore, we observed that blockage of CCR5 receptor might drive unique effects in duodenal immunity, including a paradoxical increase on T-cell activation in relation with improvement of T-cell differentiation, that could be explained by higher tissue penetration and by class-dependent effects independent of HIV suppression (Table 2).

**Table 2:** Summary of the effects of combined CCR5/Integrase Inhibitors-based regimen vs. CCR5 inhibition on intestinal T cell subpopulations.

Combined CCR5/integrase inhibitors-based regimen		CCR5 inhibitor-based regimen	
Effect	Suggested mechanism	Effect	Suggested mechanism
Better CCR5^+^/CD4^+^ T-cell restoration in rectum and duodenum	*Synergism/Class effects*	Paradoxical increase of CD8^+^ naïve T-cell activation in duodenum	*CCR5 signaling*
			*Cell mediated t*
			*Penetration effec*
Greater reduction of duodenal CD8+ T cell infiltration	*CCR5* ^*+*^ */CD4* ^*+*^ *T-cell restoration/Decrease in antigen burden*	Improvement of T-cell maturation (naïve/memory ratio) in duodenum	*Secondary to CD8* ^*+*^ *naïve T-cell activation*
			*Cell mediated*
Greater decrease in sCD14 levels	*Secondary to CCR5* ^*+*^ */CD4* ^*+*^ *T-cell restoration*		
Greater reduction of cell-associated HIV DNA levels in duodenum	*Synergism/Class effects*		

As shown by Cassol et al., compared to the large intestine, greater immunological abnormalities exist in the small intestine (i.e., more severe CD4^+^ T-cell depletion, greater CD8^+^ T-cell expansion and CD8^+^ T-cell activation), as well as poorer recovery of the mucosal immune parameters following ART initiation (i.e., persistence of CD4^+^ T-cell depletion)[51]. These observations are largely replicated in the present study. Indeed, there is increasing awareness than the small intestine could represent a portal for systemic inflammation and a preferential site of HIV persistence [19,52,53].

Antiretroviral intensification has been proposed as a strategy to target residual viral persistence and immune dysfunction. This approach has been tested in a number of clinic trials with conflicting results [54–59]. However, unlike the design of our present study which enrolled treatment naïve patients, intensification studies have been conducted in the setting of well-controlled HIV infection, and limited experience exists in naïve patients initiating first-line ART with this combination of ART classes. In our study, several findings support a beneficial role of a quadruple CCR5 and integrase-inhibitor containing first-line regimen on the gut immunity, especially in the duodenum. First, subjects with MVC+RAL showed significantly better CCR5^+^/CD4^+^ T-cell restoration in the gut, consistent with that observed in an uncontrolled trial of 15 subjects initiating quintuple ART during acute HIV infection [60]. Depletion of CD4^+^/CCR5^+^ subset is a key feature of HIV-associated mucosal dysfunction, which probably contributes to increased microbial translocation and contributes to pathogenesis of HIV-associated systemic immune activation. Second, the CCR5 and integrase inhibitors combination determined a greater decrease in sCD14 levels. Although the differences were modest, this is one of the best characterized marker of activation of innate immunity and small increments of this biomarker have shown to independently predict mortality, suggesting that quadruple ART-based regimens could improve long-term clinical outcomes [1–3]. Third, we found evidence (with a borderline statistical significance) that the quadruple ART regimen elicited a greater reduction of cell-associated HIV DNA levels in duodenum, indicating that this combination of agents could potentially contribute to a multipronged strategy to reduce the HIV reservoir. As we did not observed differences in HIV RNA levels in plasma or tissue among treatment groups, these differences might be secondary to better immune restoration rather than a direct consequence of greater HIV suppression. Given that gut biopsies were obtained with a 9-mont time interval we cannot rule out, however, that these effects were driven by a faster decline of HIV transcription with the quadruple regimen at earlier time-points, which represents a limitation to the study design. Conflicting data on the effects of intensification on markers of mucosal immunity and viral persistence have been reported in studies in ART-treated patients with poor CD4^+^ T-cell recovery, which represent a different immunological environment than in the present study. Notwithstanding, our findings replicated the results of a controlled trial in patients with poor CD4^+^ T-cell recovery in whom intensification with MVC lead to increased rectal CD4^+^ T-cell activation, as we found in both groups of patients treated with MVC [61]. In contrast, in a different controlled trial, intensification with RAL did not affect rectal T-cell activation [62], but affected viral production [63]. The inconsistent findings between our study and previous publications potentially resides with this distinction in design, including the site of GALT mucosa examined [57].

We also observed that MVC, either within a standard triple regimen or within a quadruple regimen, could exert unique effects on gut mucosa. T-cell differentiation was profoundly altered at baseline in the CD8^+^ T-cell subset. Subjects in both MVC-containing regimens experienced a significant improvement in naïve/memory CD8^+^ T-cell ratio, reaching nearly normal levels. In contrast, in the NNRTI cohort, the distribution of T-cell maturational subsets worsened during treatment for unknown reasons. This MVC-mediated improvement of intestinal T-cell differentiation correlated well with activation of the naïve subset. Activation of rectal T-cells has previously been described in a controlled trial with MVC in ART-treated patients with poor immunological recovery [61], and attributed to MVC-mediated increases in CCR5 ligands, which may activate T-cells via alternative chemokine receptors. This hypothesis was supported by an increase of circulating levels of the CCR5 ligand MIP-1β that may also signal via alternative co-receptors such as CCR1 on neutrophils in monocytes/macrophages [64]. Alternatively, a partial agonist effect of MVC on CCR5 might result in intracellular signaling pathways and explain the increment of T-cell activation observed among the study subjects receiving MVC. We recently demonstrated this in an uncontrolled study in ART treated individuals, in whom a ten-day intensification with MVC resulted in activation of NF-κB and target genes [65]. In the present study, these MVC-mediated increases of mucosal T-cell activation appeared to reverse the abnormal T-cell differentiation, as we observed strong correlations in rectum and duodenum between the distribution of CD8^+^ T maturational subsets and the numbers of naive CD8^+^ HLA-DR^+^CD38^+^ T-cells. We think that the beneficial effects of MVC on mucosal immunity could also explain the improvement of gut microbiota composition recently reported in a mouse model of obesity [66], which could reciprocally contribute to enhanced intestinal T-cell reconstitution.

We found evidence that the distinct observed effects of MVC correlated with a higher drug distribution to the gut. Compared to EFV, MVC penetration in the gut mucosa was in rectum approximately ten-fold higher, and in duodenum two-fold higher. Compared to RAL, MVC penetration was in rectum approximately forty-fold higher, and in duodenum thirty-fold higher. It is unclear, however, whether in vitro IC50 can be compared to tissue IC50 due to protein-binding effects, and this suboptimal penetration did not correlate with higher cell-associated HIV RNA or DNA tissue level. The higher MVC concentration appeared particularly relevant in duodenum, where MVC concentrations significantly correlated with several markers of the mucosal immunity, including the CD4/CD8 ratio, activated T-cells and zonulin-1. Paradoxically, MVC concentrations were lower in duodenum than in rectum, at levels below the IC50 for MIP-1β inhibition, suggesting that part of the beneficial effect on duodenal mucosa was mediated by alternative CCR5 signaling rather than favorable pharmacokinetic conditions. Although data on antiretroviral tissue levels in clinical studies are limited, it has been recently demonstrated that, compared to peripheral blood, drug concentrations achieved in the gut or in the lymph nodes are lower, and this impaired drug penetration allows persistent HIV replication in lymphatic tissues [18].

There are limitations to the current study that should be appreciated in order to reduce the risk of over-interpretation of and/or misperceptions about the presented data. First, the lack of a comparator group receiving FTC/TDF+RAL, hinders the dissection of the effects driven by each individual drug or by the use of a quadruple regimen in in the group receiving MVC+RAL. In a previous randomized clinical trial that we performed in a comparable treatment naïve patient population comparing RAL vs. NNRTI, each in combination with FTC/TDF, we also observed greater reduction of CD8^+^ T-cell density and sCD14 in the RAL compared with NNRT arm [25]. So, it is possible that some of the effects observed in the quadruple ART regimen relative to the MVC arm, such as the greater normalization of numbers of mucosal CD4^+^CCR5^+^ T-cells, were attributable to some extent by the unique effects of RAL on viral kinetics [27]. Second, given the small sample size the statistical power for some comparisons is limited, as indicated, for example, by the fact that overall differences in duodenal HIV DNA decay by treatment group was statistically significant (P = 0.008), but the P values drop to border-line values in the pairwise comparisons (MVC vs. MVC+RAL, P = 0.067) and our findings warrant confirmation in larger studies. Third, we acknowledge that that we analyzed a many different exploratory parameters with a limited sample size, so the risk of detection of less robust associations is notable and must be taken into consideration when interpreting our results during the planning of any future larger clinical trials testing the observations reported in this randomized controlled trial.

In conclusion, our data describe potential beneficial consequences of combined CCR5 and integrase inhibitor-based first-line ART regimens and highlight the impact of MVC on mucosal immunity, particularly in the duodenum, that may be due to a higher tissue penetration and by class-dependent effects. These findings suggest an opportunity for targeting mucosal immune dysfunction and chronic inflammation with regimens designed to achieve greater concentrations within lymphatic tissues, and provide rationale for expanding the armamentarium available to target the gut in future HIV reservoir reduction strategies.

## Supporting Information

S1 Checklist CONSORT Checklist. (DOCX)

S1 Table Effects of three ART regimens on lymphocyte subsets in blood. (DOCX)

S2 Table Effects of three ART regimens on lymphocyte subsets in rectum. (DOCX)

S3 Table Effects of three ART regimens on lymphocyte subsets in duodenum. (DOCX)

S4 Table Effects of three ART regimens on cell-associated HIV RNA and HIV DNA. (DOCX)

S5 Table Effects of three ART regimens on immune activation, bacterial translocation, and epithelial tight junction. (DOCX)

S6 Table Correlations between plasma biomarkers and drug concentrations. (DOCX)

S1 Fig Gating strategy. The single-cell suspensions were stained with Aqua-viability dye and QuantumDot655 anti-CD45RA (clone MEM-56) from Invitrogen (Carlsbad, California, USA); PacBlue-anti-CD3 (clone UCHT1) and fluorescein isothiocyanate-anti-human leukocyte antigen-DR (clone L243) from Biolegend (San Diego, California, USA); ECD-anti-CD4 (clone SFCI12T4D11) from Beckman- Coulter (Brea, California, USA); and PE-anti-CD38 (clone HB7), PE-Cy7-anti-CCR7 (clone 3D12), and APC-H7-anti-CD8 (clone SK1) from Becton-Dickinson (San Jose, California, USA). Gating strategy included using an FMO (fluorescence- minus-one) to determine the cut-off for positive cells for CCR7, CD38, and HLA-DR for each run. Lymphocyte maturational subsets are defined as naive (CD45RA+/CCR7+), central memory (CD45RA-/CCR7+), effector memory (CD45RA-/CCR7-), or RA+ memory (CD45RA+/CCR7-). T-cell activation is defined as co-expression of HLA-DR and CD38 on respective lymphocyte population.
(JPG)

S2 Fig Immunohistochemistry analysis. The primary antibodies were polyclonal anti-CD3 rabbit serum (Dako Inc., Carpinteria, California, USA) and monoclonal anti-CD4 or CD8 mouse serum (Leica Micreosystems, Buffalo Grove, Illinois, USA). Binding of CD3 and CD4 or CD8 receptors were detected simultaneously using Alexafluor 488-labeled polyclonal goat antirabbit IgG (Molecular Probes, Eugene, Oregon, USA) and Alexafluor 568- labeled polyclonal goat antimouse IgG (Molecular Probes). The numbers of positive cells were counted by a single observer and presented as cells/mm^2^ of lamina propria or intraepithelial regions (above the basement membrane) of rectal and duodenal mucosa.
(JPG)
