# Correction: Effects of Combined CCR5/Integrase Inhibitors-Based Regimen on Mucosal Immunity in HIV-Infected Patients Naïve to Antiretroviral Therapy: A Pilot Randomized Trial

**DOI:** 10.1371/journal.ppat.1005540

**Published:** 2016-03-25

**Authors:** Sergio Serrano-Villar, Talia Sainz, Zhong-Min Ma, Netanya S. Utay, Tae-Wook Chun, Surinder Mann, Angela D. Kashuba, Basile Siewe, Anthony Albanese, Paolo Troia-Cancio, Elizabeth Sinclair, Anoma Somasunderam, Tammy Yotter, Steven G. Deeks, Alan Landay, Richard B. Pollard, Christopher J. Miller, Santiago Moreno, David M. Asmuth

The fifth author's name is incorrect. The correct name is Tae-Wook Chun. The correct citation is: Serrano-Villar S, Sainz T, Ma Z-M, Utay NS, Chun T-W, Mann S, et al. (2016) Effects of Combined CCR5/Integrase Inhibitors-Based Regimen on Mucosal Immunity in HIV-Infected Patients Naïve to Antiretroviral Therapy: A Pilot Randomized Trial. PLoS Pathog 12(1): e1005381. doi:10.1371/journal.ppat.1005381
