# Correction: Effects of Combined CCR5/Integrase Inhibitors-Based Regimen on Mucosal Immunity in HIV-Infected Patients Naïve to Antiretroviral Therapy: A Pilot Randomized Trial

**DOI:** 10.1371/journal.ppat.1006368

**Published:** 2017-05-03

**Authors:** Sergio Serrano-Villar, Talia Sainz, Zhong-Min Ma, Netanya S. Utay, Tae Wook-Chun, Surinder Mann, Angela D. Kashuba, Basile Siewe, Anthony Albanese, Paolo Troia-Cancio, Elizabeth Sinclair, Anoma Somasunderam, Tammy Yotter, Steven G. Deeks, Alan Landay, Richard B. Pollard, Christopher J. Miller, Santiago Moreno, David M. Asmuth

There are two errors in Figs 7 and 9. In Fig 7A and 7B, the Y-axis are given as percentage of plasma/tissue concentrations. However, the plasma/tissue concentration ratios were calculated as the ratio of plasma concentration measured in ng/mL over the tissue concentration measured in ng/mg. Hence, the tissue concentrations have been converted to ng/mL assuming a tissue density of 1.06 g/mL, and Y-axis are given as the plasma/tissue concentration ratio. As shown in the original figure, maraviroc reached the highest distribution to rectum and duodenum (all P<0.005). These changes do not alter the findings derived from the original figure.

**Fig 7:**
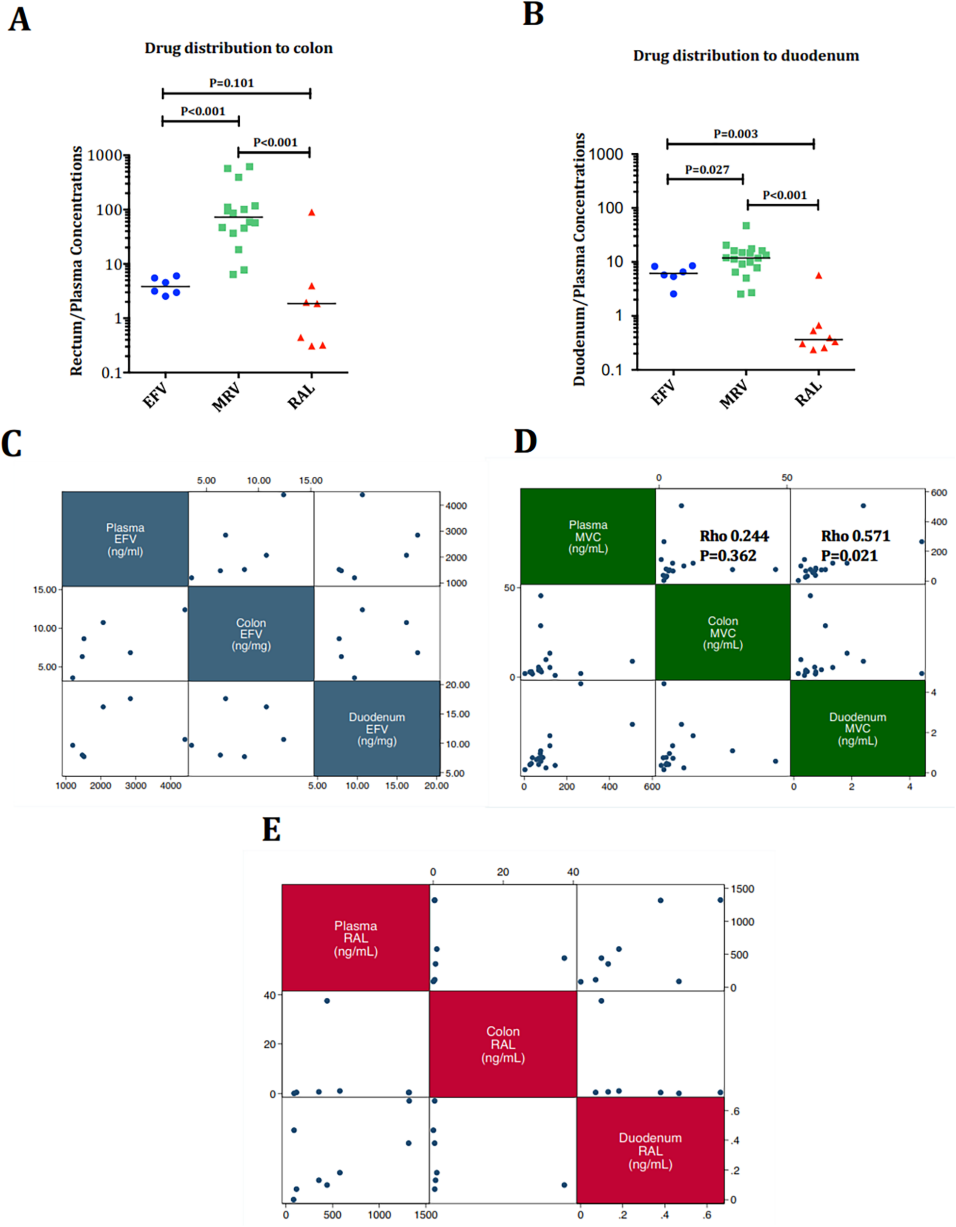
Tissue drug distribution. Panels A-B. Rectum/plasma (A) and duodenum/plasma (B) drug concentration ratios. The tissue concentrations converted to ng/mL assuming a tissue density of 1.06 g/mL. Maraviroc reached the highest distribution to rectum and duodenum (all P<0.005). Panel C-E. Correlations between plasma, rectum and duodenal levels of EFV (C),MVC(D) and RAL (E). MVC plasma levels correlated better with tissue levels than RAL or EFV.

**Fig 9:**
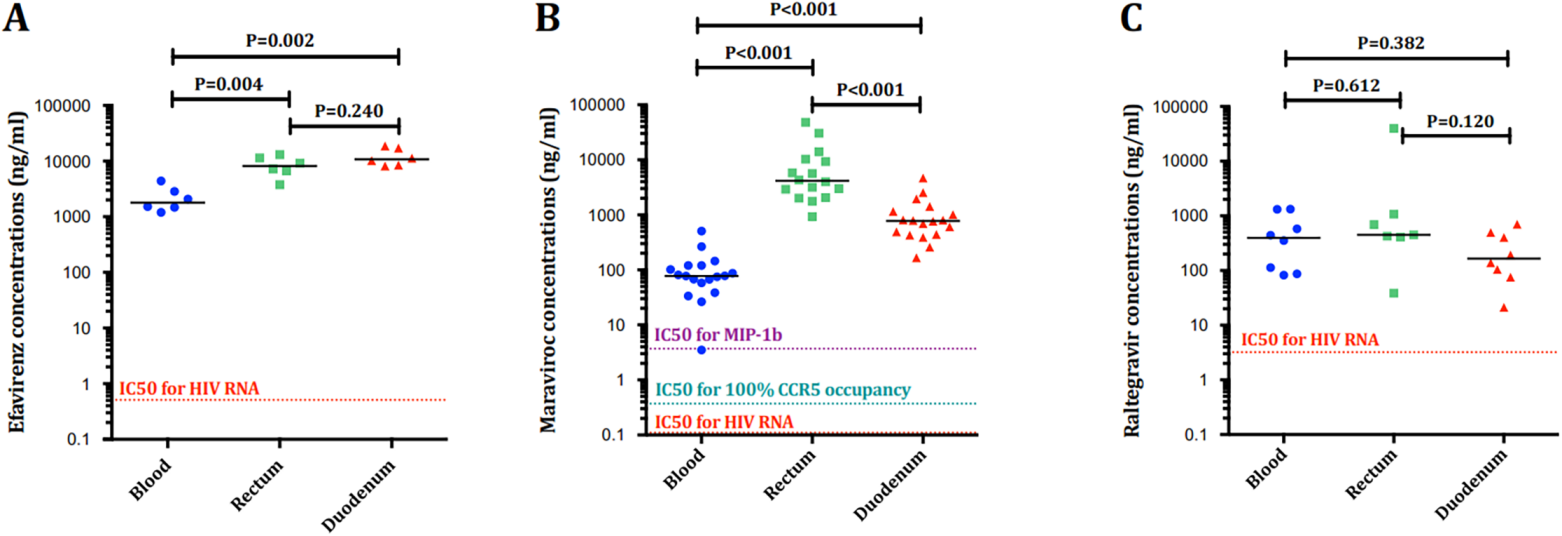
**Absolute concentrations in each compartment of efavirenz (A), maraviroc (B) and raltegravir (C) related to the IC50 for HIV-1 replication.** The tissue concentrations converted to ng/mL assuming a tissue density of 1.06 g/mL.

In Fig 9B and 9C, the Y-axis and IC50 values are given as ng/mL. However, the raltegravir and maraviroc concentrations are plotted as ng/mg. Hence, the values have been converted to ng/mL assuming a tissue density of 1.06 g/mL. Consequently, all the drugs reached concentrations above the IC50 in all compartments, in contrast which what is shown in the original figure, in which raltegravir levels are below the IC50 in rectum and duodenum. As shown in the previous version of the figure, we found higher median values of maraviroc in duodenum than in rectum [4157 ng/ml (2273–10041) vs. 778 (438–1223), P<0.001), respectively]. Maraviroc concentrations reached values above the IC50 for all MIP-1b inhibition, 100% CCR5 occupancy and HIV RNA.

The figure legends have also been modified to reflect the changes. The authors confirm that these changes do not alter their conclusions.
