# Supplementary material for: Effects of Combined CCR5/Integrase Inhibitors-Based Regimen on Mucosal Immunity in HIV-Infected Patients Naïve to Antiretroviral Therapy: A Pilot Randomized Trial
Source: PLoS Pathog. 2016 Jan 21;12(1):e1005381. doi: 10.1371/journal.ppat.1005381 (PMC4721954; doi:10.1371/journal.ppat.1005381)
Supplement: S1 Table — (DOCX) [file ppat.1005381.s002.docx]

**Table S1. Effects of three ART regimens on lymphocyte subsets in blood.**

|  | **1: NNRTI** | | **2: MVC** | | **3: MVC+RAL** | |
| --- | --- | --- | --- | --- | --- | --- |
|  | **Baseline mean (95% CI)** | **Mean delta change (95% CI)** | **Baseline mean (95% CI)** | **Mean delta change (95% CI)** | **Baseline mean (95% CI)** | **Mean delta change (95% CI)** |
| **CD4+ T-cells/mm^3^** | 393 (264, 523) | 194 (57, 330) | 428 (342, 514) | 221 (149, 293) | 515 (361, 669) | 231 (134, 327) |
|  |  |  |  |  |  |  |
| **%CD4+ T-cells** | 27.0 (18.9, 35.0) | 14.8 (8.1, 21.4) | 26.8 (22.5, 31.0) | 13.8 (8.1, 19.4) | 35.3 (30.3, 40.2) | 7.0 (2.1, 11.8) |
| **CCR5+** | 39.6 (241, 55.1) | -24.2 (-40.5, -7.9) | 45.5 (33.0, 58.1) | -13.4 (-28.6, 1.8) | 29.3 (21.1, 37.6) | 3.4 (-8.5, 15.2) |
| **HLADR+CD38+** | 20.7 (8.2, 33.3) | -13.9 (-27.8, -3.0) | 9.4 (6.4, 12.5) | -2.0 (-6.4, 2.4) | 9.4 (6.2, 12.7) | -2.7 (-6.2, 0.8) |
| ***Maturational subsets*** |  |  |  |  |  |  |
| **Naïve** | 36.2 (27.2, 45.3) | 7.1 (-5.9, 20.1) | 44.0 (33.9, 54.2) | 4.0 (-3.9, 11.9) | 42.1 (34.4, 49.8) | 4.1 (-1.4, 9.8) |
| **T_CM_** | 30.4 (22.5, 38.3) | -7.9 (-18.9, 3.0) | 22.4 (18.8, 26.0) | -4.0 (-8.9, 0.8) | 22.2 (15.7, 28.8) | -5.3 (-11.5, 0.9) |
| **T_EM_** | 27.0 (21.3, 32.8) | -2.4 (-8.7, 3.8) | 25.5 (18.5, 32.4) | -4.1 (-10.1, 1.8) | 26.3 (21.1, 31.5) | -3.9 (-9.4, 1.6) |
| **T_EMRA_** | 6.3 (2.8, 9.9) | 3.3 (1.0, 7.5) | 8.1 (2.6, 13.9) | 4.2 (2.3, 6.2) | 9.4 (5.9, 12.8) | 5.2 (-1.2, 11.5) |
| **Naive/Memory** | 0.64 (0.39, 0.88) | 0.17 (-0.55, 0.90) | 0.97 (0.55, 1.39) | 0.11 (-0.28, 0.50) | 0.79 (0.53, 1.0) | -0.13 (-0.08, 0.33) |
| **Naïve HLA-DR+CD38+** | 3.6 (0.6, 6.6) | -2.2 (-4.4, 0.1) | 1.0 (0.6, 1.3) | 1.1 (-1.0, 3.3) | 1.7 (0.3, 3.1) | -0.4 (-1.9, 1.2) |
|  |  |  |  |  |  |  |
| **%CD8+ T-cells** | 64.9 (58.1, 71.7) | -12.9 (-17.5, -8.2) | 64.5 (59.6, 69.4) | -12.1 (-16.6, -7.6) | 57.9 (53.9, 62.0) | -6.5 (-11.7, -1.4) |
| **HLADR+CD38+** | 60.5 (46.5,74.5) | -26.3 (-35.5, -20.1) | 44.8 (38.0, 54.5) | -23.7 (-32.3, -15.1) | 52.1 (41.3, 62.9) | -25.3 (-35.2, -15.3) |
| ***Maturational subsets*** |  |  |  |  |  |  |
| **Naïve** | 18.4 (13.4, 23.4) | 6.6 (-5.7, 18.8) | 19.8 (12.0, 27.6) | 15.8 (7.7, 23.9) | 20.7 (15.0, 26.4) | 8.8 (2.8, 14.7) |
| **T_CM_** | 6.6 (3.3, 9.8) | -5.0 (-8.7, -1.3) | 4.4 (2.0, 6.9) | -2.8 (-5.3, -0.4) | 2.9 (0.8, 5.1) | -2.1 (-4.0, -0.2) |
| **T_EM_** | 34.4 (22.3, 46.6) | -14.8 (-25.6, -4.1) | 31.5 (22.4, 40.7) | -13.4 (-21.6, -5.2) | 22.9 (16.1, 29.8) | -12.6 (-19.7, -5.5) |
| **T_EMRA_** | 40.7 (31.3, 50.0) | 13.2 (-1.6, 28.0) | 44.2 (31.2, 57.2) | 0.49 (-9.3, 10.3) | 53.4 (45.4, 61.4) | 5.9 (-2.4, 14.2) |
| **Naïve/Memory** | 0.24 (0.15, 0.32) | 0.16 (-0.11, 0.43) | 0.28 (0.12, 0.44) | 0.32 (0.15, 0.49) | 0.27 (0.19, 0.36) | 0.20 (0.03, 0.37) |
| **Naïve HLA-DR+CD38+** | 8.9 (2.2, 15.6) | -6.7 (-10.9, -2.5) | 2.9 (1.3, 4.5) | 0.2 (-3.0, 3.5) | 5.7 (1.6, 9.8) | -3.4 (-7.4, 0.5) |
| **Memory HLA-DR+CD38+** | 52.8 (42.8, 62.9) | -19.4 (-26.5, -12.3) | 41.3 (32.3, 50.4) | -19.6 (-31.7, -7.6) | 44.2 (34.8, 53.5) | -16.4 (-27.4, -5.4) |
|  |  |  |  |  |  |  |
| **CD4/CD8 ratio** | 0.45 (0.26, 0.63) | 0.43 (0.24, 0.62) | 0.43 (0.34, 0.52) | 0.36 (0.23, 0.49) | 0.62 (0.50, 0.74) | 0.28 (0.03, 0.52) |
| *Reported means and 95% CI represent point estimates computed by linear mixed models with a random effect for each patient before log-transformation.* | | | | | | |
