# Supplementary material for: Effects of Combined CCR5/Integrase Inhibitors-Based Regimen on Mucosal Immunity in HIV-Infected Patients Naïve to Antiretroviral Therapy: A Pilot Randomized Trial
Source: PLoS Pathog. 2016 Jan 21;12(1):e1005381. doi: 10.1371/journal.ppat.1005381 (PMC4721954; doi:10.1371/journal.ppat.1005381)
Supplement: S2 Table — (DOCX) [file ppat.1005381.s003.docx]

**Table S2. Effects of three ART regimens on lymphocyte subsets in rectum.**

|  | **1: NNRTI** | | **2: MVC** | | **3: MVC+RAL** | |
| --- | --- | --- | --- | --- | --- | --- |
|  | **Baseline mean (95% CI)** | **Mean delta change (95% CI)** | **Baseline mean (95% CI)** | **Mean delta change (95% CI)** | **Baseline mean (95% CI)** | **Mean delta change (95% CI)** |
| **%CD4+ T-cells** | 21.5 (12.7, 30.4) | 20.6 (16.3, 24.9) | 17.9 (11.4, 24.5) | 18.7 (13.7, 23.7) | 19.4 (16.1, 22.7) | 18.7 (14.1, 23.1) |
| **CCR5+** | 85.8 (81.3, 90.2) | -10.6 (-24.5, 3.4) | 88.2 (78.9, 97.5) | -3.5 (-13.4, 6.5) | 82.8 (73.2, 92.3) | 0.6 (-8.7, 10.0) |
| **HLADR+CD38+** | 39.4 (28.7, 50.1) | -11.5 (-21.8, -1.2) | 39.4 (32.6, 46.2) | -12.6 (-22.5, -2.6) | 31.9 (24.4, 39.3) | -2.1 (-7.9, 3.8) |
| ***Maturational subsets*** |  |  |  |  |  |  |
| **Naïve** | 10.2 (4.6, 15.9) | 1.7 (-11.1, 14.6) | 10.8 (3.7, 17.8 ) | -1.1 (-10.4, 8.3) | 9.8 (4.2, 15.5) | 2.2 (-2.9, 7.3) |
| **T_CM_** | 35.8 (24.8, 46.8) | -6.6 (-21.2, 8.1) | 39.7 (26.5, 52.9) | -14.8 (-32.9, 3.1) | 34.8 (23.4, 46.3) | -2.3 (-21.2, 16.7) |
| **T_EM_** | 48.6 (39.3, 57.9) | 5.8 (-14.0, 25.5) | 45.9 (30.1, 61.8) | 11.5 (-9.8, 32.9) | 51.4 (39.7, 63.2) | -2.2 (-22.2, 17.8) |
| **T_EMRA_** | 5.4 (1.7, 9.1) | -0.8 (-5.2, 3.5) | 3.5 (0.8, 6.3) | 4.5 (0.7, 8.5) | 3.9 (1.8, 5.9) | 2.8 (-0.4, 6.1) |
| **Naive/Memory** | 0.12 (0.04, 0.21) | 0.06 (-0.18, 0.30) | 0.14 (0.03, 0.25) | -0.03 (-0.17, 0.11) | 0.12 (0.04, 0.19) | 0.03 (-0.03, 0.09) |
| **Naïve HLA-DR+CD38+** | 6.6 (1.1, 12.1) | -3.2 (-8.0, 1.5) | 5.9 (1.2, 10.7) | -1.2 (-7.0, 4.6) | 4.3 (2.4, 6.2) | 2.3 (-1.1, 5.8) |
|  |  |  |  |  |  |  |
| **%CD8+ T-cells** | 70.8 (61.3, 80.3) | -23.1 (-18.3, -17.8) | 72.3 (64.9, 79.8) | -20.8 (-27.2,-14.4) | 73.3 (68.7, 78.0) | -21.5 (-25.2, -17.8) |
| **HLADR+CD38+** | 76.7 (66.5, 86.9) | -20.4 (-28.2, -12.5) | 70.9 (63.3, 78.4) | -21-7 (-35.4,-7.9) | 72.7 (66.2, 79.2 ) | -20.0 (-28.6, -11.2) |
| ***Maturational subsets*** |  |  |  |  |  |  |
| **Naïve** | 14.1 (4.8, 23.5) | -1.9 (-12.0, 8.2) | 11.4 (6.2, 16.7) | 6.6 (-5.8, 19.0) | 10.8 (5.4, 16.1) | 8.1 (-1.1, 17.3) |
| **T_CM_** | 23.5 (13.1, 33.9) | -8.4 (-21.2, 4.5) | 21.3 (11.5, 31.0) | -9.1 (-23.2, 5.0) | 22.2 (9.6, 34.9) | -6.1 (-21.1, 8.8) |
| **T_EM_** | 51.2 (33.8, 64.6) | -0.1 (-15.9, 15.8) | 51.4 (39.8, 63.0) | -2.9 (-25.0, 19.1) | 52.3 (37.8, 66.7) | -11.9 (-28.5, 4.7) |
| **T_EMRA_** | 11.2 (5.1, 17.3) | 10.3 (-1.8, 22.5) | 15.9 (7.2, 24.6) | 5.5 (-6.9, 17.8) | 14.8 (9.4, 20.2) | 9.8 (-3.8, 23.5) |
| **Naive/Memory** | 0.20 (0.03, 0.37) | -0.06 (-0.23, 0.12) | 0.14 (0.07, 0.21) | 0.13 (-0.08,0.33) | 0.13 (0.06, 0.20) | 0.15 (-0.02, 0.33) |
| **Naïve HLA-DR+CD38+** | 12.5 (3.3, 21.7) | -5.3 (-13.2, 2.7) | 9.4 (4.6, 14.1) | 3.3 (-6.4, 13.1) | 8.5 (3.8, 13.2) | 5.6 (-2.2, 13.3) |
| **Memory HLA-DR+CD38+** | 65.4 (55.7, 75.4) | -15.8 (-27.3, -4.2) | 61.8 (54.7, 68.9) | -25.2 (-40.9, -9.5) | 59.4 (51.3, 67.6) | -16.1 (-27.9, -4.2) |
|  |  |  |  |  |  |  |
| **CD4/CD8 ratio** | 0.36 (0.15, 0.57) | 0.62 (0.43, 0.81) | 0.26 (0.12, 0.39) | 0.57 (0.34, 0.79) | 0.28 (0.22, 0.33) | 0.48 (0.34, 0.61) |
| *Reported means and 95% CI represent point estimates computed by linear mixed models with a random effect for each patient before log-transformation.* | | | | | | |
