# Supplementary material for: Effects of Combined CCR5/Integrase Inhibitors-Based Regimen on Mucosal Immunity in HIV-Infected Patients Naïve to Antiretroviral Therapy: A Pilot Randomized Trial
Source: PLoS Pathog. 2016 Jan 21;12(1):e1005381. doi: 10.1371/journal.ppat.1005381 (PMC4721954; doi:10.1371/journal.ppat.1005381)
Supplement: S3 Table — (DOCX) [file ppat.1005381.s004.docx]

**Table S3. Effects of three ART regimens on lymphocyte subsets in duodenum.**

|  | **1: NNRTI** | | **2: MVC** | | **3: MVC+RAL** | |
| --- | --- | --- | --- | --- | --- | --- |
|  | **Baseline mean (95% CI)** | **Mean delta change (95% CI)** | **Baseline mean (95% CI)** | **Mean delta change (95% CI)** | **Baseline mean (95% CI)** | **Mean delta change (95% CI)** |
| **%CD4+ T-cells** | 11.6 (2.4, 20.7) | 10.5 (7.7, 13.4) | 8.3 (4.7, 11.9) | 10.9 (6.7, 15.1) | 10.1 (6.7, 13.4) | 14.1 (7.3, 20.9) |
| **CCR5+** | 90.0 (84.7, 95.4) | -1.8 (-6.0, 2.4) | 94.6 (93.0, 96.2) | -3.9 (-8.9, 1.1) | 85.3 (76.7, 94.0) | 4.0 (-5.1, 13.1) |
| **HLADR+CD38+** | 56.0 (45.2, 66.7) | -5.8 (-14.9, 3.2) | 49.7 (41.4, 58.0) | -11.1 (-26.2, 4.0) | 51.0 (44.8, 57.2) | -8.4 (-19.5, 2.7) |
| ***Maturational subsets*** |  |  |  |  |  |  |
| **Naïve** | 10.6 (3.7, 17.5) | -4.2 (-11.3, 2.9) | 7.6 (2.9, 12.3) | 0.5 (-6.2, 7.2) | 5.0 (2.1, 8.0) | 4.7 (-1.6, 11.0) |
| **T_CM_** | 32.9 (20.1, 45.7) | -6.7 (-18.0, 4.5) | 36.2 (23.8, 48.6) | -10.1 (-26.0, 5.9) | 29.8 (16.1, 43.5) | -2.0 (-22.9, 18.9) |
| **T_EM_** | 51.6 (39.8, 63.4) | 11.0 (2.3, 19.7) | 51.7 (37.8, 65.7) | 8.0 (-12.1, 28.1) | 60.9 (45.8, 76.1 ) | -5.5 (-27.9, 17.0) |
| **T_EMRA_** | 4.9 (2.6, 7.3) | -0.1 (-3.5, 3.3) | 4.6 (2.1, 7.0) | 1.5 (-3.2, 6.3) | 4.3 (1.4, 7.1) | 3.2 (-2.2, 8.5) |
| **Naive/Memory** | 0.14 (0.03, 0.24) | -0.07 (-0.17, 0.04) | 0.09 (0.03, 0.15) | 0.01 (-0.07, 0.09) | 0.05 (0.02, 0.09) | 0.06 (-0.02, 0.15) |
| **Naïve HLA-DR+CD38+** | 9.6 (3.0, 16.2) | -4.5 (-11.1, 2.0) | 5.0 (1.4, 8.6) | 1.2 (-3.5, 5.8) | 4.3 (1.7, 6.9) | 3.7 (-1.9, 9.2) |
|  |  |  |  |  |  |  |
| **%CD8+ T-cells** | 83.2 (72.3, 94.1) | -9.9 (-13.5, -6.4) | 85.5 (81.8, 89.3) | -14.7 (-19.8, -9.7) | 83.5 (77.8, 89.2) | -13.2 (-19.6, -6.8) |
| **HLADR+CD38+** | 67.4 (55.7, 79.3) | -6.2 (-16.0, 3.7) | 57.5 (48.4, 66.7) | -11.9 (-27.1, 3.4) | 67.8 (58.9, 76.6) | -12.9 (-27.8, 1.9) |
| ***Maturational subsets*** |  |  |  |  |  |  |
| **Naïve** | 11.4 (3.5, 19.4) | -5.1 (-12.1, 1.9) | 9.1 (6.0, 12.2) | 4.9 (-3.4, 13.3) | 6.7 (3.5, 9.9) | 10.2 (-0.9, 21.3) |
| **T_CM_** | 18.9 (9.1, 28.8) | -5.3 (-14.3, 3.7) | 21.3 (14.7, 27.9) | -5.0 (-14.7, 4.5) | 18.5 (10.8, 26.3) | -5.4 (14.0, 3.3) |
| **T_EM_** | 59.1 (46.5, 71.7) | 2.4 (-8.1, 13.0) | 54.6 (48.1, 61.1) | -3.3 (-20.5, 13.3) | 61.8 (52.3, 71.3) | -15.4 (-35.2, 4.4) |
| **T_EMRA_** | 10.5 (6.8, 14.2) | 7.9 (-0.8, 16.6) | 16.0 (7.8, 24.3) | 2.3 (-9.9, 14.6) | 13.3 (9.0, 17.6) | 10.2 (-2.3, 22.7) |
| **Naive/Memory** | 0.15 (0.03, 0.27) | -0.08 (-0.19, 0.03) | 0.10 (0.07, 0.14 ) | 0.09 (-0.04, 0.23) | 0.07 (0.04, 0.11) | 0.17 (-0.2, 0.37) |
| **Naïve HLA-DR+CD38+** | 10.8 (2.9, 18.6) | -5.3 (-12.3, 1.7) | 7.4 (4.2, 10.6) | 2.7 (-3.4, 8.8) | 5.7 (2.7, 8.6) | 8.5 (-1.1, 18.0) |
| **Memory HLA-DR+CD38+** | 57.2 (47.7, 66.8) | -2.6 (-12.4, 7.1) | 49.9 (42.7, 56.9) | -20.5 (-36.1, -4.8) | 53.9 (41.5, 66.2) | -9.3 (-26.1, 7.4) |
|  |  |  |  |  |  |  |
| **CD4/CD8 ratio** | 0.18 (0.02, 0.35) | 0.19 (0.11, 0.26) | 0.10 (0.05, 0.15) | 0.20 (0.08, 0.32) | 0.12 (0.08, 0.17) | 0.28 (0.08, 0.48) |
|  |  |  |  |  |  |  |
| **Immunohistochemistry** |  |  |  |  |  |  |
| **CD4+ T-cells/mm^2^** | 108 (-20, 236) | 119 (-7, 245) | 130 (44, 215) | 24 (-44, 92) | 96 (19, 173) | 89 (51,127) |
| **CD8+ T-cells/mm^2^** | 1418 (1099, 1739) | -333 (-698, 31) | 1441 (1051, 1833) | -626 (-1025, -228) | 1507 (1173, 1841) | -670 (-955, -384) |
| *Reported means and 95% CI represent point estimates computed by linear mixed models with a random effect for each patient before log-transformation.* | | | | | | |
