# Supplementary material for: Effects of Combined CCR5/Integrase Inhibitors-Based Regimen on Mucosal Immunity in HIV-Infected Patients Naïve to Antiretroviral Therapy: A Pilot Randomized Trial
Source: PLoS Pathog. 2016 Jan 21;12(1):e1005381. doi: 10.1371/journal.ppat.1005381 (PMC4721954; doi:10.1371/journal.ppat.1005381)
Supplement: S4 Table — (DOCX) [file ppat.1005381.s005.docx]

**Table S4. Effects of three ART regimens on cell-associated HIV RNA and HIV DNA.**

|  | **1: NNRTI** | | **2: MVC** | | **3: MVC+RAL** | |
| --- | --- | --- | --- | --- | --- | --- |
|  | **Baseline mean (95% CI)** | **Mean delta change (95% CI)** | **Baseline mean (95% CI)** | **Mean delta change (95% CI)** | **Baseline mean (95% CI)** | **Mean delta change (95% CI)** |
| **HIV RNA** |  |  |  |  |  |  |
| **Blood - HIV RNA (copies/CD4+ T cell)** | 52047 (1290, 102804) | -52037 (-102786, -1290) | 19809 (4483, 35135) | -19804 (-35127, -4482) | 26851 (9432, 44270) | -26848 (-44266, -9428) |
| **Rectum - HIV RNA**  **(copies/CD4+ T cell)** | 91955 (13407, 170502) | -91944 (-170476, -13411) | 24671 (1041, 48301) | -24670 (-48300, -1041) | 154052 (-64455, 372559) | -154051 (-372559, 64456) |
| **Duodenum - HIV-RNA**  **(copies/10^6^ cells)** | 8463 (-6048, 22974) | -8459 (-22971, 6053) | 1145 (-410, 2701) | -1144 (-2699, 410) | 5682 (-3876, 15239) | -5680 (-15234, 3874) |
| **HIV DNA** |  |  |  |  |  |  |
| **Blood - HIV DNA (copies/10^6^ cells)** | 4405 (432, 8379) | -1330 (-5354, 2693) | 3247 (1752, 4741) | -1709 (-2845, -573) | 4861 (1259, 8462) | -2500 (-4868, -132) |
| **Rectum - HIV DNA**  **(copies/10^6^ cells)** | 433 (48, 817) | -53 (-302, 195) | 174 (-18.9, 369) | -142 (-349, 65) | 738 (263, 1213) | -392 (-661, -122) |
| **Duodenum - HIV-DNA**  **(copies/10^6^ cells)** | 388 (-76, 851) | -325 (-701, 51) | 36 (6, 67) | -16 (-40, 8) | 938 (89, 1787) | -846 (-1721, 30) |
| *Reported means and 95% CI represent point estimates computed by linear mixed models with a random effect for each patient before log-transformation.* | | | | | | |
