# Supplementary material for: Effects of Combined CCR5/Integrase Inhibitors-Based Regimen on Mucosal Immunity in HIV-Infected Patients Naïve to Antiretroviral Therapy: A Pilot Randomized Trial
Source: PLoS Pathog. 2016 Jan 21;12(1):e1005381. doi: 10.1371/journal.ppat.1005381 (PMC4721954; doi:10.1371/journal.ppat.1005381)
Supplement: S5 Table — (DOCX) [file ppat.1005381.s006.docx]

**Table S5. Effects of three ART regimens on immune activation, bacterial translocation, and epithelial tight junction.**

|  | **1: NNRTI** | | **2: MVC** | | **3: MVC+RAL** | |
| --- | --- | --- | --- | --- | --- | --- |
|  | **Baseline mean (95% CI)** | **Mean delta change (95% CI)** | **Baseline mean (95% CI)** | **Mean delta change (95% CI)** | **Baseline mean (95% CI)** | **Mean delta change (95% CI)** |
| **Interleukin-6 (pg/ml)** | 4.3 (1.0, 7.5.) | -0.2 (-0.6, 0.3) | 3.1 (1.8, 4.3) | -0.06 (-0.3, 0.1) | 4.7 (-0.1, 9,5) | -0.3 (-0.8, 0.2) |
| **Soluble sCD14 (ug/ml)** | 2.6 (2.2, 3.0) | 0.09 (-0.28, 0.46) | 2.4 (2.2, 2.7) | -0.3 (-0.5, -0.07) | 2.1 (1.9, 2.4) | -0.3 (-0.5, -0.1) |
| **Lipoteichoic acid*** | 0.19 (0.12, 0.26) | -0.05 (-0.08, -0.02) | 0.19 (0.14, 0.24) | -0.07 (0.003) | 0.15 (-0.09, 0.02) | -0.01 (-0.06, 0.04) |
| **Zonulin-1 (ng/ml)** | 19.0 (14.2, 23.8) | 4.8 (-2.3, 11.9) | 29.5 (12.8, 46.1) | -8.0 (-17.8, 1.7) | 30.2 (18.1, 42.2) | 6.9 (2.0, 11.7) |
| *ELISA optical density values  *Reported means and 95% CI represent point estimates computed by linear mixed models with a random effect for each patient before log-transformation.* | | | | | | |
