# Supplementary material for: Effects of Combined CCR5/Integrase Inhibitors-Based Regimen on Mucosal Immunity in HIV-Infected Patients Naïve to Antiretroviral Therapy: A Pilot Randomized Trial
Source: PLoS Pathog. 2016 Jan 21;12(1):e1005381. doi: 10.1371/journal.ppat.1005381 (PMC4721954; doi:10.1371/journal.ppat.1005381)
Supplement: S6 Table — (DOCX) [file ppat.1005381.s007.docx]

**Table S6. Correlations between plasma biomarkers and drug concentrations.**

|  | **Concentrations** | **Interleukin-6** | | **Soluble CD14** | | **Lipoteichoic acid** | | **Zonulin-1** | |
| --- | --- | --- | --- | --- | --- | --- | --- | --- | --- |
|  | **Median (P25-P75)** | **Rho** | **P value** | **Rho** | **P value** | **Rho** | **P value** | **Rho** | **P value** |
| **Efavirez** |  |  |  |  |  |  |  |  |  |
| **Plasma (ng/ml)** | 1796.1 (1473.9, 2847.5) | -0.543 | 0.266 | 0.371 | 0.469 | 0.657 | 0.156 | 0.257 | 0.623 |
| **Rectum** | 7.7 (6.3, 10.7) | -0.542 | 0.266 | 0.086 | 0.872 | 0.429 | 0.397 | 0.543 | 0.266 |
| **Duodenum (ng/mg)** | 10.2 (8.0, 16.2) | -0.600 | 0.201 | 0.257 | 0.623 | 0.371 | 0.469 | 0.029 | 0.957 |
|  |  |  |  |  |  |  |  |  |  |
| **Maraviroc** |  |  |  |  |  |  |  |  |  |
| **Plasma (ng/ml)** | 77.6 (58.0, 120.3) | 0.097 | 0.721 | -0.194 | 0.471 | 0.0 | 1.0 | 0.235 | 0.389 |
| **Rectum** | 3.9 (2.3, 9.2) | -0.065 | 0.812 | -0.259 | 0.333 | 0.176 | 0.513 | -0.073 | 0.787 |
| **Duodenum (ng/mg)** | 0.7 (0.4, 1.1) | 0.103 | 0.704 | -0.671 | 0.004 | 0.056 | 0.837 | 0.209 | 0.438 |
|  |  |  |  |  |  |  |  |  |  |
| **Raltegravir** |  |  |  |  |  |  |  |  |  |
| **Plasma (ng/ml)** | 397.0 (99.9, 946.9) | 0.036 | 0.939 | -0.500 | 0.253 | 0.143 | 0.760 | 0.893 | **0.006** |
| **Rectum (ng/mg)** | 0.4 (0.3, 1.0) | -0.464 | 0.294 | -0.464 | 0.294 | 0.213 | 0.644 | 0.0 | 1.0 |
| **Duodenum (ng/mg)** | 0.2 (0.1, 0.4) | -0.143 | 0.759 | 0.143 | 0.759 | -0.143 | 0.759 | 0.464 | 0.294 |
