# Supplementary figures and images for: Effects of Combined CCR5/Integrase Inhibitors-Based Regimen on Mucosal Immunity in HIV-Infected Patients Naïve to Antiretroviral Therapy: A Pilot Randomized Trial
Source: PLoS Pathog. 2016 Jan 21;12(1):e1005381. doi: 10.1371/journal.ppat.1005381 (PMC4721954; doi:10.1371/journal.ppat.1005381)

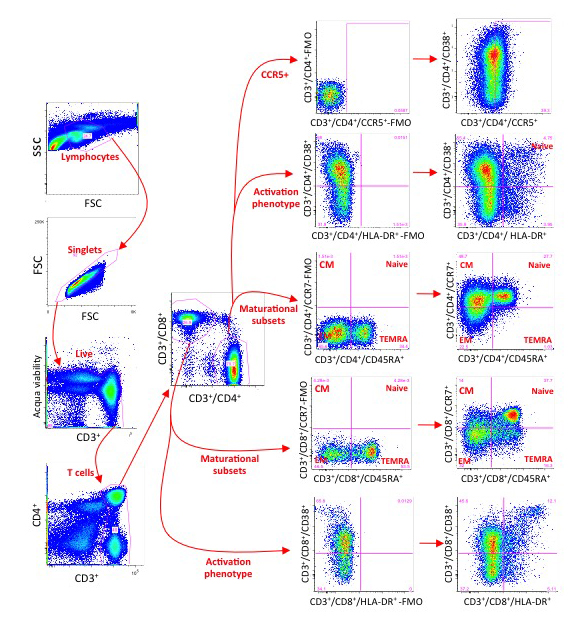

Supplement: S1 Fig — The single-cell suspensions were stained with Aqua-viability dye and QuantumDot655 anti-CD45RA (clone MEM-56) from Invitrogen (Carlsbad, California, USA); PacBlue-anti-CD3 (clone UCHT1) and fluorescein isothiocyanate-anti-human leukocyte antigen-DR (clone L243) from Biolegend (San Diego, California, USA); ECD-anti-CD4 (clone SFCI12T4D11) from Beckman- Coulter (Brea, California, USA); and PE-anti-CD38 (clone HB7), PE-Cy7-anti-CCR7 (clone 3D12), and APC-H7-anti-CD8 (clone SK1) from Becton-Dickinson (San Jose, California, USA). Gating strategy included using an FMO (fluorescence- minus-one) to determine the cut-off for positive cells for CCR7, CD38, and HLA-DR for each run. Lymphocyte maturational subsets are defined as naive (CD45RA+/CCR7+), central memory (CD45RA-/CCR7+), effector memory (CD45RA-/CCR7-), or RA+ memory (CD45RA+/CCR7-). T-cell activation is defined as co-expression of HLA-DR and CD38 on respective lymphocyte population. (JPG) [file ppat.1005381.s008.jpg]

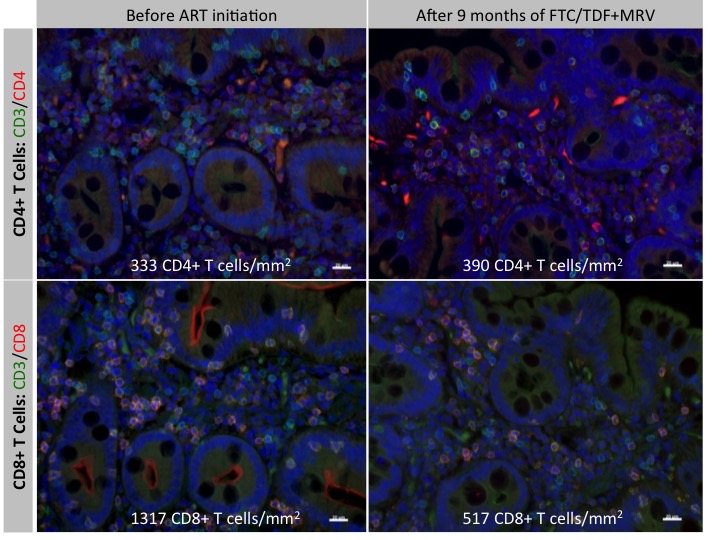

Supplement: S2 Fig — The primary antibodies were polyclonal anti-CD3 rabbit serum (Dako Inc., Carpinteria, California, USA) and monoclonal anti-CD4 or CD8 mouse serum (Leica Micreosystems, Buffalo Grove, Illinois, USA). Binding of CD3 and CD4 or CD8 receptors were detected simultaneously using Alexafluor 488-labeled polyclonal goat antirabbit IgG (Molecular Probes, Eugene, Oregon, USA) and Alexafluor 568- labeled polyclonal goat antimouse IgG (Molecular Probes). The numbers of positive cells were counted by a single observer and presented as cells/mm2 of lamina propria or intraepithelial regions (above the basement membrane) of rectal and duodenal mucosa. (JPG) [file ppat.1005381.s009.jpg]
